# Hassall’s corpuscles with cellular-senescence features maintain IFNα production through neutrophils and pDC activation in the thymus

**DOI:** 10.1093/intimm/dxy073

**Published:** 2018-12-10

**Authors:** Jianwei Wang, Miho Sekai, Takeshi Matsui, Yosuke Fujii, Mitsuru Matsumoto, Osamu Takeuchi, Nagahiro Minato, Yoko Hamazaki

**Affiliations:** 1 Department of Immunology and Cell Biology, Graduate School of Medicine, Kyoto University, Kyoto, Japan; 2 Laboratory of Immunobiology, Graduate School of Medicine, Center for iPS Cell Research and Application (CiRA), Kyoto University, Kawahara-cho, Shogoin, Sakyo-ku, Kyoto, Japan; 3 Laboratory for Skin Homeostasis, RIKEN Center for Integrative Medical Sciences (IMS), Yokohama, Kanagawa, Japan; 4 Division of Molecular Immunology, Institute for Enzyme Research, Tokushima University, Tokushima, Japan; 5 Department of Medical Chemistry, Graduate School of Medicine, Kyoto University, Kyoto, Japan

**Keywords:** Hassall’s bodies, cell senescence, type I interferon, mTEC, thymic epithelial cells

## Abstract

Hassall’s corpuscles (HCs) are composed of cornifying, terminally differentiated medullary thymic epithelial cells (mTECs) that are developed under the control of Aire. Here, we demonstrated that HC-mTECs show features of cellular senescence and produce inflammatory cytokines and chemokines including CXCL5, thereby recruiting and activating neutrophils to produce IL-23 in the thymic medulla. We further indicated that thymic plasmacytoid dendritic cells (pDCs) expressing IL-23 receptors constitutively produced *Ifna*, which plays a role in single positive (SP) cell maturation, in an *Il23a*-dependent manner. Neutrophil depletion with anti-Ly6G antibody injection resulted in a significant decrease of *Ifna* expression in the thymic pDCs, suggesting that thymic neutrophil activation underlies the *Ifna* expression in thymic pDCs in steady state conditions. A New Zealand White mouse strain showing HC hyperplasia exhibited greater numbers and activation of thymic neutrophils and pDCs than B6 mice, whereas *Aire*-deficient B6 mice with defective HC development and SP thymocyte maturation showed significantly compromised numbers and activation of these cells. These results collectively suggested that HC-mTECs with cell-senescence features initiate a unique cell activation cascade including neutrophils and pDCs leading to the constitutive IFNα expression required for SP T-cell maturation in the thymic medulla.

## Teaser

Hassall’s corpuscles activate neutrophils to stimulate pDCs in the thymus

## Introduction

The thymus provides a critical microenvironment for T-cell development and selection. Thymic epithelial cells (TECs) are a major component of thymic stroma, produce various cytokines and chemokines to support T-cell proliferation, differentiation, migration and survival, and present self-peptides in the context of major histocompatibility complexes (MHCs) for selection. The thymic medulla plays a crucial role in inducing negative selection and regulatory T cell (T_reg_) generation (1), in which various types of stromal cells and antigen-presenting cells (APCs) are involved. Among these cells, medullary TECs (mTECs), partly under the control of Aire, contribute to this process by displaying numerous tissue-specific antigens (TSAs) and by producing various chemokines to promote the recruitment of positively selected thymocytes and APCs into the medulla (1). Conventional dendritic cells (cDCs), plasmacytoid DCs (pDCs) and B cells recruited from the periphery also present blood-borne self-antigens to developing thymocytes in the medulla (2). Additionally, positively selected CD4 or CD8 single positive (SP) thymocytes in the medulla further proliferate to increase the clone size and undergo final maturation to be licensed for functional responsiveness in the periphery (3). Therefore, the thymic medulla provides a microenvironment critical for establishing central T-cell self-tolerance and completion of T-cell development.

Recent studies have revealed that the development and maturation of mTECs depend on lymphoid-lineage cells providing tumor necrosis factor receptor (TNFR) superfamily signals, including receptor activator of nuclear factor kappa-B (NF-κB) (RANK)-, lymphotoxin beta receptor (LTβR)- and CD40-related signals, via NF-κB activation (4, 5). cDC maturation is compromised in *RelB*- and *Traf6*-knockout mice (6, 7), and a recent report indicated that circulating naive B cells are activated by CD4^+^ T cells via CD40 signaling to express Aire and up-regulate the expression of MHC class II (MHC II) molecules (8). These results suggest that the NF-κB-related signaling axis commonly underlies the development and maturation of various APCs in the medulla. On the other hand, type I interferons are expressed in the thymic medulla under steady state conditions in humans and mice, with IFNα and IFNβ constitutively expressed in pDCs and mTECs, respectively (9–12). Indeed, interferon-inducible genes are detected in SP thymocytes and mTECs, and type I interferons play a role in SP thymocyte maturation (13, 14). However, little is known about the mechanisms of type I interferon induction in the thymic medulla.

The thymic medulla includes unique structures called Hassall’s bodies/corpuscles (HCs), which were first described in humans and consist of mTECs undergoing cornification (15). Although the structures are less prominent in rodents, recent studies reported comparable structures in mice according to the expression of several molecules, including involucrin, keratin 1 (K1), keratin 10 (K10), desmoglein 3 (Dsg3) and tight-junction molecules, which are normally expressed in cornifying epidermal keratinocytes (16–20). Lineage-tracing analysis indicated that HCs consist of MHC II^low^ post-Aire mTECs (21–23), and, consistently, involucrin^+^ mTEC clusters are significantly reduced in the thymus of *Aire*^*–/–*^ mice (20). Furthermore, the deficiency of LTβR- and transforming growth factor-β-related signals in mice results in the decrease and increase of HCs, respectively (17, 24). Functionally, myasthenia gravis (MG) patients with thymic hyperplasia exhibit increased numbers of HCs with high levels of chemokine ligand (CCL) 21 expression, implying a possible role for HCs in recruiting pathogenic T cells and/or naive B cells into the thymic medulla (25). Additionally, human HCs produce thymic stromal lymphopoietin (TSLP) and might contribute to the promotion of T_reg_ production through DC activation *in vitro* (26). However, the number of T_regs_ is grossly normal in Aire- and LTβR-deficient mice (27, 28); therefore, aside from these sporadic findings, the exact function of HCs remains a matter of debate.

In this study, we found that skin-specific retroviral-like aspartic protease SASPase (*Asprv1*), which is expressed during the cornification process of epidermal keratinocytes (29, 30), was expressed in HC-mTECs at a high level. Taking advantage of this, we examined the molecular features of isolated HC-mTECs using enhanced green fluorescence protein (EGFP)/*Asprv1* knock-in (KI) mice. We demonstrated that these HC-mTECs displayed features of cell senescence, expressed pro-inflammatory genes including IL-1 family cytokines and neutrophil-recruiting chemokines and induced the constitutive activation of innate immune cells, such as neutrophils and pDCs, which produced IL-23 and IFNα, respectively, in the thymic medulla. Consistently, mature SP thymocytes were significantly reduced in *Il23a*- and *Aire*-deficient mice. These results suggest that HCs contribute to the constitutive activation of neutrophils and pDCs in the thymic environment to produce IFNα, thus possibly promoting thymocyte maturation.

## Methods

### Mice

C57BL/6N (B6) and New Zealand White (NZW) mice were obtained from SLC Japan (Hamamatsu, Japan). SASP-EGFP-KI mice were obtained from RIKEN-CLST (accession no. CDB1022K, Matsui *et al.*, in preparation). Briefly, the exon of SASPase/*ASPRV1* was replaced with an *EGFP*-*Neo* cassette consisting of the *EGFP* gene and the neomycin resistance gene (*Neo*) flanked by *loxP* sequences. *Aire*^*–/–*^, *Il23a*^–/–^ mice were described previously (31, 32). *Ifnar*2^–/–^ mice were purchased from B&K Universal (Grimston, UK). All animals were maintained in specific pathogen-free conditions at the Kyoto University Laboratory Animal Center in accordance with university guidelines. Mice between 6 and 8 weeks of age were used unless otherwise mentioned. This study was performed in accordance with the principles expressed in the Declaration of Helsinki and approved by the Animal Research Committee, Graduate School of Medicine, Kyoto University (MedKyo16596).

### Antibodies and other reagents

The following fluorescent or biotinylated antibodies and reagents were used for staining mouse cells and tissues unless otherwise stated: CD3 (clone 145-2C11; BioLegend, San Diego, CA, USA), CD4 (clone GK1.5; BioLegend), CD8 (clone 53-6.7; BioLegend), CD14 (clone sa2-8; eBioscience; Thermo Fisher Scientific, Waltham, MA, USA), CD25 (clone PC61; eBioscience), CD45 (clone 30-F11; eBioscience), CD69 (clone H1.2F3; eBioscience), CD80 (clone 16-10A1, BioLegend), CCR9 (clone CW-1.2; eBioscience), epithelial cell adhesion molecule (EpCAM; clone G8.8; BioLegend), Ly51 (clone 6C3; eBioscience), Ly6G (clone 1A8; BioLegend), MHC II (clone M5/114.15.2; BioLegend), PDCA-1 (clone 129c; eBioscience), Ter119 (clone Ter119; BioLegend), IL-23R (clone 12B2B64; BioLegend), CD11b (clone M1/70; BioLegend), Gr1 (clone RB6-8C5; BioLegend), Qa2(clone 695h1-9-9; BioLegend), Ki67 (clone olA15; eBioscience), Dsg3 [clone AK18; a gift from Prof. Masayuki Amagai, Keio University (Tokyo, Japan)] (33), *Ulex europaeus* agglutinin-1 (Vector Laboratories, Burlingame, CA, USA), K10 (rabbit polyclonal; Covance Laboratories, Raleigh, NC, USA), pan-keratin (rabbit polyclonal; DAKO, Tokyo, Japan), involucrin (rabbit polyclonal; BioLegend), claudin-3 (rabbit polyclonal; Novus Biologicals, Littleton, CO, USA), claudin-4 (rabbit polyclonal; Invitrogen, Carlsbad, CA, USA) and SASPase [rabbit polyclonal (29)]. Anti-Aire Armenian hamster monoclonal antibody (clone HF108M5; IgG) was generated in Matsumoto’s Laboratory by immunization with the peptides corresponding to the C-terminal portion of mouse Aire (34). Anti-SASPase rat monoclonal antibody (clone mG2-C) was generated by the immunization of rats with glutathione *S*-transferase-mouse SASPase fusion protein and screened with maltose-binding proteins-mouse SASPase and was gift from KAN Research Institute. The C-terminal fragment of *Clostridium perfringens* enterotoxin (C-CPE) was prepared as previously described (35). InVivoPlus anti-mouse Ly6G and InVivoPlus rat IgG2a isotype control antibodies were purchased from BioXCell and used for the intra-peritoneal injection *in vivo* study.

### Immunostaining

Immunostaining was performed as previously described (36). Tissues were embedded in optimal cutting temperature compound (Sakura, Tokyo, Japan), frozen in liquid nitrogen and cut into sections (6 µm) on a Leica CM3050S cryostat (Leica Biosystems, Wetzlar, Germany). Sections were fixed in 95% ethanol for 30 min at 4°C, followed by incubation in 100% acetone for 1 min at room temperature. To detect EGFP signals, tissues were fixed with 2% paraformaldehyde (Nacalai, Kyoto, Japan) and cryoprotected in 30% sucrose before embedding. Fixed samples were blocked with 1% bovine serum albumin/phosphate-buffered saline (PBS) overnight at 4°C, followed by incubation with primary antibodies for 1 h and secondary reagents for 30 min at room temperature. Stained tissues were mounted with Mowiol 4-88 (Calbiochem, San Diego, CA, USA), and images were captured on a Axiovert 200M microscope (Carl Zeiss, Oberkochen, Germany) using Axio Vision release 4.8 acquisition software (Carl Zeiss).

### Cytospin and Giemsa staining

Sorted CD45^+^CD11b^+^Ly6G^+^ neutrophils from mouse thymus and peripheral blood (PB) were cytospun and stained with May–Grunwald Giemsa solution (Muto Pure Chemicals, Tokyo, Japan) and examined using a light microscope (Olympus Optical Company, Tokyo, Japan).

### SA-β-Gal staining

SA-β-Gal staining was performed using a kit from Cell Signaling Technology (Danvers, MA, USA) according to the manufacturer’s protocol. Briefly, cryosections of B6 and NZW thymus tissues were dried for 10 min and fixed with fixative solution (Cell Signaling Technology) for between 10 and 15 min at room temperature. Slides were rinsed twice with 1× PBS, and after the last wash, 1 ml staining solution [1 mg ml^−1^ 5-bromo-4-chloro-3-inolyl-β-d-galactoside in dimethylformamide (20 mg ml^−1^ stock), 40 mM citric acid/sodium phosphate (pH 6.0), 5 mM potassium ferrocyanide, 5 mM potassium ferricyanide, 150 mM NaCl, and 2 mM MgCl_2_] was added. The slides were incubated at 37°C overnight. After incubation, the cells were washed twice with PBS and photographed.

### Cell isolation and culture

TEC isolation was performed as previously described (37). Briefly, thymi were dissected into small specimens and sedimented, and the thymocyte-rich supernatant was roughly removed. The sediments were treated with LiberaseTM (Roche, Basel, Switzerland), and the cell suspensions were blocked with an anti-FcRIIb (clone 2.4G2; TONBO, San Diego, CA, USA) antibody and incubated with anti-CD45 microbeads. The CD45^+^ fraction was further depleted using an AutoMACS system (Miltenyi Biotec, Bergisch Gladbach, Germany), multi-color stained using various antibodies and analyzed using a FACS Canto II system (BD Biosciences) equipped with FACS DIVA (BD Biosciences). The CD45^−^EpCAM^+^ fraction was defined as the TEC fraction, and mTECs and cortical TECs (cTECs) were further divided as UEA1^+^Ly51^−^ and UEA-1^−^Ly51^+^ cells, respectively. To analyze the proportions of various cell types in the thymus, enzyme-treated cell suspensions were re-mixed with the thymocyte-rich supernatant and analyzed. For the sorting and culture of thymic pDCs, Thy1.2^+^ cells were depleted using an AutoMACS system (Miltenyi Biotec), and CD11c^low^B220^+^ cells were sorted using a FACSAriaII system (BD Biosciences). Sorted pDCs (2 × 10^5^) were cultured for 12 h in 96-well plates in medium containing RPMI-1640 supplemented with 10% fetal calf serum (FCS), 100 U ml^−1^ penicillin, 100 µg ml^−1^ streptomycin and 50 µM mercaptoethanol in the presence of CpG (10 ng ml^−1^) and in the presence or absence of IL-23 (100 ng ml^−1^) (BioLegend). For neutrophil isolation, Thy1.2^+^ cells were depleted using an AutoMACS system (Miltenyi Biotec), and CD45^+^CD11b^+^Ly6G^+^ cells were sorted using a FACSAriaII system (BD Biosciences). For *in vitro* neutrophil cultures, sorted neutrophils (1 × 10^5^) were cultured for 12 h in 96-well plates in medium containing RPMI-1640 supplemented with 10% FCS, 100 U ml^−1^ penicillin, 100 µg ml^−1^ streptomycin and 50 µM mercaptoethanol in the presence of CpG (1 µg ml^−1^), lipopolysaccharide (LPS; 1 µg ml^−1^) and ploy(I:C) (100 µg ml^−1^). Targeted cell purity was ≥95%.

### Neutrophil depletion in vivo

NZW mice received purified anti-mouse Ly6G antibody (BioXCell, West Lebanon, NH, USA) administration (200 µg per shot) intra-peritoneally every third day for 2 weeks. The mice were analyzed on the next day of the last shot. Mice that received rat IgG2a isotype were used as control groups.

### Quantitative reverse transcription–PCR analysis

Total RNA was isolated from freshly prepared and sorted cells using TRIzol reagent (Thermo Fisher Scientific), reverse transcribed with SuperScript III using oligo(dT)12-18 primers (Invitrogen) and amplified with SYBR Green I (Roche). *Actb* was used as an internal control. All the primers were synthesized by Invitrogen. The primer sets used are listed in Supplementary Table 1.

### Immunohistochemistry of human thymus tissue

Human thymus tissue samples were purchased from US Biomax (Derwood, MD, USA). Paraffin-enabled tissue sections were antigen-retrieved using a prestige stainless steel pressure cooker for 5 min at 121°C. After antigen retrieval, endogenous peroxidase activity was blocked by 0.3% H_2_O_2_ in methyl alcohol for 30 min, and slides were washed with PBS. Primary antibodies were subsequently applied overnight at 4°C, followed by washing with PBS. Secondary antibodies were diluted 1:300 in PBS for 40 min, and the following antibodies were used for human thymus immunohistochemistry staining: CD15 (1:200; clone Leu-M1; Abcam, Cambridge, MA, USA), CD66b (1:50; rabbit polyclonal; Abcam) and pan-keratin (1:50; clone AE1/AE3; DAKO, Carpinteria, CA, USA).

### Statistical analysis

All statistical analyses were performed using a two-tailed student’s *t*-test for comparison between two groups or one-way analysis of variance (ANOVA) with Tukey *post hoc* test for multiple comparison. A *P* value <0.05 was considered to indicate a significant difference.

## Results

### Elevated expression of SASPase characterizes rare HC-mTECs

**Fig. 1.**
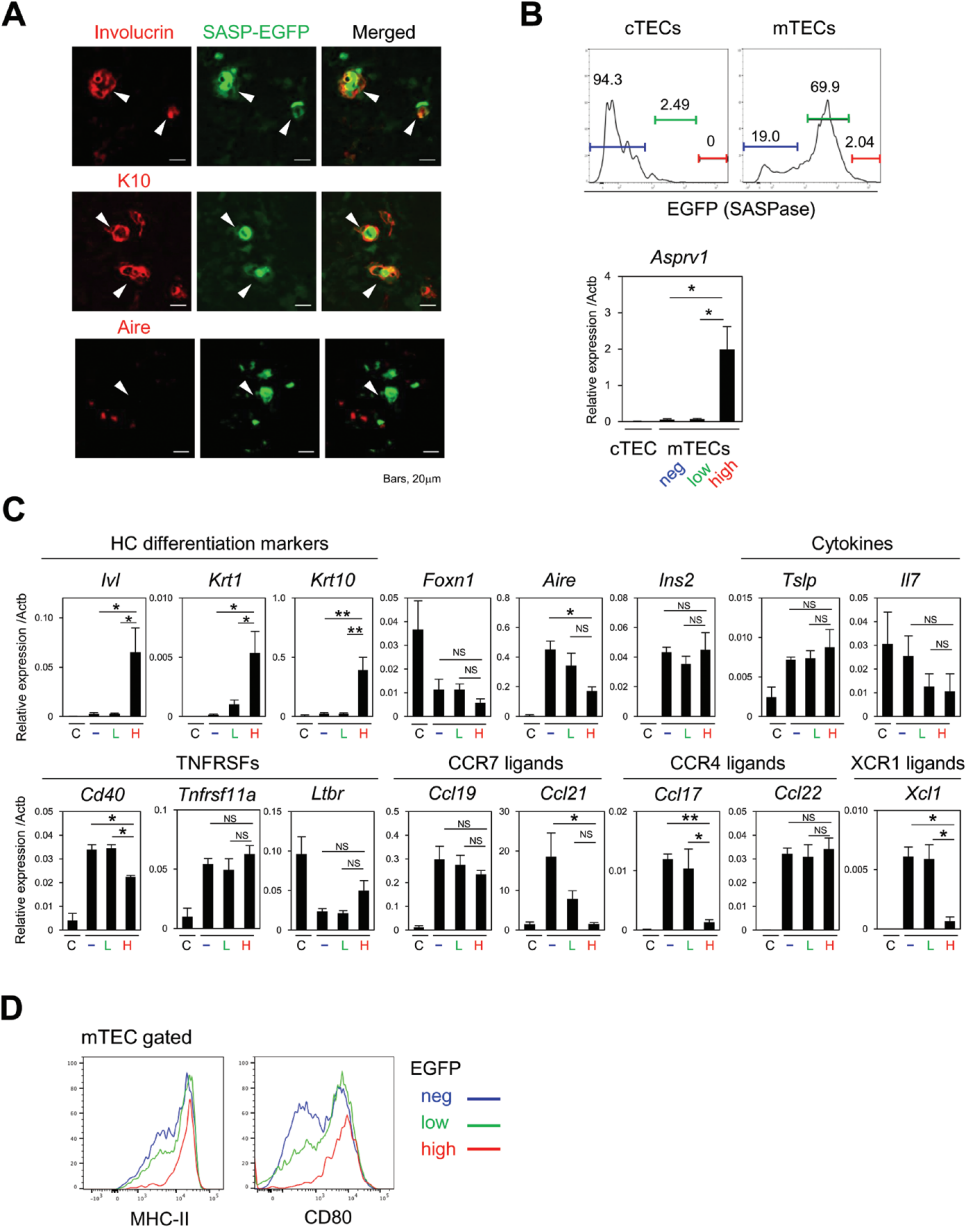
SASPase-EGFP^**high**^ cells show characteristics of HC-mTECs. (A) The thymus of EGFP/*Asprv1*-KI mice (SASP-EGFP-KI mice) was two-color stained with involucrin, keratin 10 (K10), Aire and EGFP antibodies. Arrowheads indicate HC-like cell clusters in the medulla. Scale bars, 20 µm. (B) EGFP intensities in cTECs and mTECs from SASP-EGFP-KI mice and the percentages of EGFP-negative (neg), -low and -high cell fractions in cTECs and mTECs are shown (top). The expression of *Asprv1* transcripts relative to *Actb* in cTECs, EGFP^neg^, EGFP^low^ and EGFP^high^ mTECs was assessed by qRT–PCR (bottom). (C) The expression of the transcripts of the indicated genes relative to *Actb* in cTECs, EGFP^neg^, EGFP^low^ and EGFP^high^ mTECs were assessed by qRT–PCR. The means and SE of at least three independent experiments are shown. One-way ANOVA with Tukey *post hoc* test was performed (*P < 0.05, **P < 0.01, NS = not significant). C, cTECs; –, EGFP^neg^ mTECs; L, EGFP^low^ mTECs; H, EGFP^high^ mTECs. (D) MHC II and CD80 expression in the EGFP^neg^ (blue), EGFP^low^ (green) and EGFP^high^ (red) mTECs are shown. Data are representative of three independent experiments.

HC-mTECs undergo cornification, expressing various molecules similar to the upper layers of dermal keratinocytes, such as involucrin, K1 and K10 (16–20). SASPase (*Asprv1*), which converts profilaggrin to filaggrin to promote cornification, is expressed specifically in the stratum granulosum layers of dermal keratinocytes (29, 30). In the thymus, we found that SASPase was expressed strongly in a minor portion of mTECs (Supplementary Figure 1A). Immunostaining analysis of B6 and EGFP/*Asprv1*-knockin mice (SASP-EGFP-KI) revealed a strong EGFP signal in TEC clusters expressing SASPase, K10 and involucrin, but not Aire (Fig. 1A and Supplementary Figure 1A). Flow cytometric analysis confirmed that EGFP was expressed in the majority of the mTEC fraction (~80%), whereas EGFP expression was barely observed in cTECs (Fig. 1B and Supplementary Figure 1B). We then sorted the mTECs into three fractions based on EGFP intensity (EGFP^neg^, EGFP^low^ and EGFP^high^) and examined gene expression patterns. EGFP^high^ mTECs, which constituted ~2% of total mTECs, expressed significantly higher levels of *Asprv1* mRNA as compared with other mTEC fractions, whereas *Asprv1* mRNA in cTECs was undetectable (Fig. 1B). In agreement with immunostaining analysis, the expression of involucrin (*Ivl*), K1 (*Krt1*) and K10 (*Krt10*) mRNA was almost exclusively detected in EGFP^high^ mTECs (Fig. 1C). By contrast, EGFP^high^ mTECs showed a reduced expression of genes encoding Aire (*Aire*) as compared with the other mTEC fractions, but the expression of Aire-dependent TSA gene insulin II (*Ins2*) and forkhead box N1 (*Foxn1*) (38) was comparable with those observed in other mTECs (Fig. 1C). The expression of mTEC-related chemokines (*Ccl21*, *Ccl17* and *Xcl1*), which are involved in the recruitment of positively selected thymocytes and DCs into the medulla (39, 40), was variably reduced, whereas IL-7 (*Il7*), CCL19 (*Ccl19*), CCL22 (*Ccl22*) and TSLP (*Tslp*) expression was unchanged (Fig. 1C). The expression of CD40 (*Cd40*) was decreased, and that of RANK (*Tnfrsf11a*) and LTβR (*Ltbr*) was unchanged (Fig. 1C). Previous studies reported that K10^+^ or Involucrin^+^ HC mTECs are MHC II^low^ (17, 20–22). However, EGFP^high^ mTECs showed the highest expression of MHC II and CD80 among mTEC fractions (Fig. 1D). Thus, we further sorted for MHC II^high^ and MHC II^low^ fractions (~40% and 30%, respectively) in EGFP^high^ mTECs and investigated the gene expression. Both fractions showed significantly higher *Krt10* and lower *Aire* expression compared to EGFP^neg^ and EGFP^low^ fractions, but the tendency was more prominent in the MHC II^low^ fraction (Supplementary Figure 2A), strongly suggesting that the MHC II^high^ mTECs may differentiate into the MHC II^low^ mTECs. Immunohistochemical analysis also showed that EGFP^high^ mTECs contained MHC II^low/neg^ and MHC II^high^ mTECs and that DAPI negative EGFP^high^ dying/dead mTECs after cornification are mostly MHC II^low^. (Supplementary Figure 2B). These results indicated that a rare fraction of mTECs expressing a high level of *Asprv1* (SASP^high^) characterized HC-mTECs that are highly matured and exhibit features of cornification, but at the same time, they showed lower expression of several functional molecules expressed in the majority of mature mTECs.

### HC-mTECs show cell-senescence phenotypes and uniquely express inflammatory molecules

**Fig. 2.**
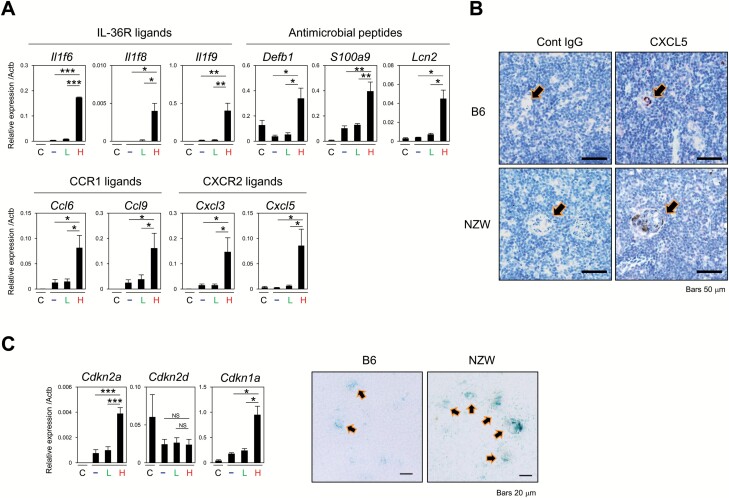
Inflammatory and senescence-related molecules are highly expressed in the SASPase(SASP)^**high**^ mTEC fraction. (A) The expression of the transcripts of the indicated genes relative to *Actb* in sorted cTECs, SASP^neg^, SASP^low^ and SASP^high^ mTECs from SASP-EGFP-KI mice was assessed by quantitative reverse transcription (qRT)–PCR. The means and SE of at least two independent experiments are shown. One-way ANOVA with Tukey *post hoc* test was performed (*P < 0.05, **P < 0.01, ***P < 0.001). (B) Paraffin-embedded thymus tissues from B6 and NZW mice were stained with an anti-CXCL5 antibody. Arrows indicate CXCL5 expression around the HC areas, with brown coloring representing positive staining. Scale bars, 50 µm. (C) The expression of the transcripts of the indicated genes relative to *Actb* was assessed by qRT–PCR (left). The means and SE of three independent experiments are shown. One-way ANOVA with Tukey *post hoc* test was performed (*P < 0.05, ***P < 0.001, NS = not significant). Cryosections of the thymus from adult B6 and NZW mice were assayed for SA-β-gal activity. Arrows indicate blue-colored β-gal-positive HC areas. Scale bars, 20 µm. Data are representative of at least two independent experiments. C, cTECs; –, SASP^neg^ mTECs; L, SASP^low^ mTECs; H, SASP^high^ mTECs.

To address possible unique functions of these HC-mTECs, we investigated genes that are highly expressed specifically in SASP^high^ HC-mTECs in SASP-EGFP-KI mice. We found that SASP^high^ mTECs exhibited an increased expression of IL-1-related pro-inflammatory cytokines of the IL-36 family (*Il1f6*, *Il1f8*, and *Il1f9*) and several chemokine genes, including ligands for CCR1 (*Ccl6* and *Ccl9*) and CXCR2 (*Cxcl3* and *Cxcl5*), compared with other mTECs and cTECs (Fig. 2A). Additionally, the expression of genes encoding antimicrobial peptides, such as defensins (*Defb1*), lipocalin 2 (*Lcn2*) and S100 proteins (*S100a9*), was markedly increased in SASP^high^ mTECs relative to that in other TECs (Fig. 2A). The expression of *Cxcl5* was prominent among chemokine genes, and CXCL5 protein was specifically detected in small HC-like mTEC clusters in the medulla of C57BL/6N (B6) mice by immunostaining (Fig. 2B). We confirmed that *Cxcl5* transcripts were significantly higher in the MHC II^high^ fraction and were more prominently up-regulated in the MHC II^low^ fraction in SASP^high^ mTECs compared to SASP^low^ and SASP^neg^ fractions (Supplementary Figure 3). Compared to B6 mice, NZW mice, a model of systemic lupus erythematosus (SLE), showed large HCs similar to those found in humans (Supplementary Figure 4A) (41). We confirmed the large HC-mTEC clusters in NZW mice also strongly expressed CXCL5 (Fig. 2B), and *Cxcl5* transcripts were highly detected in the total mTEC fraction of NZW mice compared to that of B6 mice (Supplementary Figure 4B). Because the ectopic expression of various pro-inflammatory cytokines/chemokines appeared reminiscent of a senescence-associated secretory phenotype in senescent cells (42), we examined the expression of senescence markers (43) in HC-mTECs. Sorted SASP^high^ mTECs from B6 mice showed a markedly increased expression of p16 [*cyclin-dependent kinase inhibitor* (*Cdkn*)*2a*] and p21 (*Cdkn1a*) compared with other TECs, although the expression of p19 (*Cdkn2d*) was comparable (Fig. 2C). Furthermore, HC-like clusters in the medulla of B6 mice were positive for senescence-associated beta-galactosidase (SA-β-Gal) staining, and larger SA-β-Gal-positive mTEC clusters were observed in the medulla of NZW mice relative to those observed in B6 mice (Fig. 2C). These results suggested that HC-mTECs uniquely expressed a number of pro-inflammatory cytokines, chemokines and antimicrobial peptides at steady state, possibly in relation to a senescence-associated secretory phenotype (42).

### HCs are constitutively associated with neutrophils in both mice and humans

**Fig. 3.**
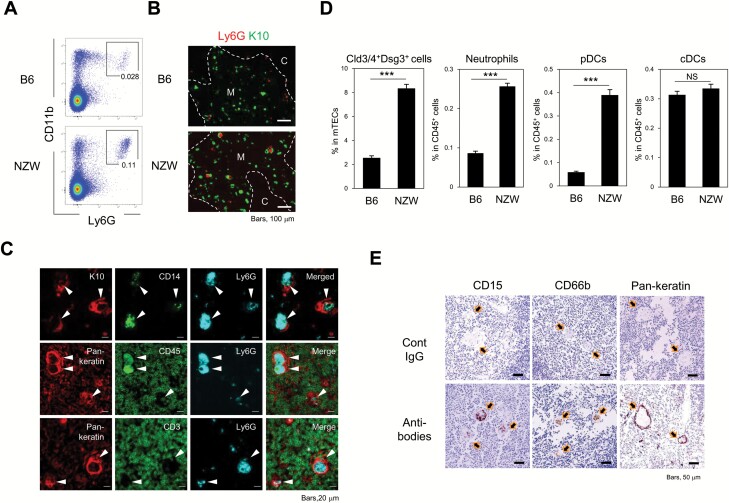
**HCs are constitutively associated with neutrophils in mice and humans.** (A) Flow cytometric analysis of total thymic cells from B6 and NZW mice using the indicated antibodies, with proportions of CD11b^+^Ly6G^+^ cells among CD45^+^ cells indicated. (B) Two-colored immunostaining of B6 and NZW mouse thymic tissues using the indicated antibodies: K10 (HC-mTECs) and Ly6G (neutrophils). Scale bars, 100 µm. (C) NZW mouse thymic tissues underwent multi-color staining using the indicated antibodies. Scale bars, 20 µm. Arrowheads indicate HC areas. (D) Percentages of thymic neutrophils (CD45^+^CD11b^+^Ly6G^+^), pDCs (CD45^+^ CD11c^low^B220^+^), cDCs (CD45^+^B220^−^CD11c^high^) and HC-mTECs (CD45^−^EpCAM^+^Cld3/4^+^Dsg3^+^) from B6 (*n* = 8) and NZW (*n* = 6) mice were examined by flow cytometry. Unpaired student’s *t*-test was performed (***P < 0.001, NS = not significant). (E) Human thymic tissues were stained with human neutrophil markers (CD15, CD66b) and an epithelial marker (pan-keratin). Arrows indicate HC areas. Scale bars, 50 µm. Data are representative of (E) two or (A–D) three independent experiments.

We then investigated the possible effect of high levels of chemokine production by HC-mTECs in thymic tissue. Among the cells in thymic tissue, *Ccr1* and CXCR2 expression was almost completely confined to neutrophils, with much lower expression observed in B cells and other myeloid cells (Supplementary Figure 5A). Additionally, sorted thymic CD45^+^CD11b^+^Ly6G^+^ cells showed segmented nuclei similar to those observed in PB, confirming that they are neutrophils (Supplementary Figure 5B). Consistent with the higher *Cxcl5* expression observed in the HC-mTECs of NZW mice relative to B6 mice (Supplementary Figure 4B), NZW mice harbored significantly more neutrophils in the thymus than B6 mice (0.11 ± 0.04% and 0.028 ± 0.007% of total thymic cells, respectively) (Fig. 3A). Immunostaining analysis revealed that Ly6G^+^ cells were mostly detected in the medullary region of both B6 and NZW mice (Fig. 3B), and that Ly6G^+^ cells co-expressing CD45 and CD14, but not CD3 or pan-keratin, were closely associated with K10^+^ HCs that apparently formed cell clusters at central regions in NZW mice (Fig. 3C). Segmented nuclei characteristic of mature neutrophils were also identified in the central regions of HCs according to hematoxylin and eosin staining (Supplementary Figure 5C). Furthermore, NZW mice showed significantly higher proportions of claudin-3,4 (Cld3/4)^+^ Dsg3^+^ HC-mTECs as well as neutrophils and pDCs, but not cDCs, compared to B6 mice (Fig. 3D and Supplementary Figure 5D). In human thymic tissues, we also found that neutrophils identified by typical markers such as CD15 and CD66b were detected as cell clusters in close association with characteristic HCs at their central regions, where pan-keratin staining was negative (Fig. 3E). These results suggested that HC-mTECs recruit neutrophils into the thymic medulla through their constitutive expression of CXCL5 in mice and humans.

### Thymic neutrophils are constitutively activated and uniquely produce IL-23

**Fig. 4.**
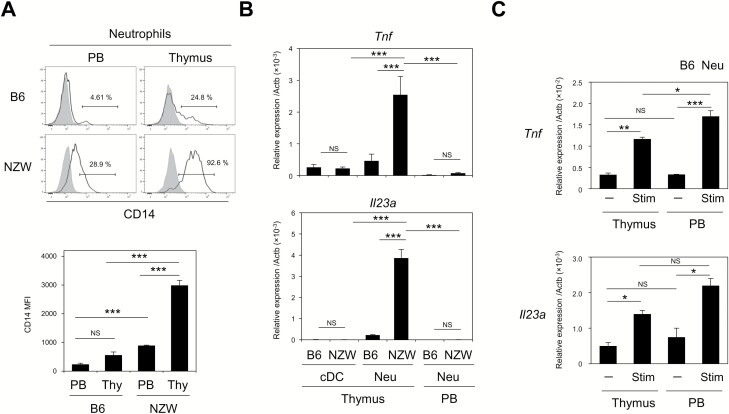
Thymic neutrophils are constitutively activated and uniquely produce IL-23. (A) CD14 expression in neutrophil fractions (CD11b^+^Ly6G^+^) from the thymus and PB of B6 and NZW mice (solid lines). Shaded regions indicate isotype control staining (upper). The mean fluorescence intensity (MFI) associated with CD14 expression in neutrophils from three independent experiments is shown. Error bars represent SE (below). (B) The expression of the *Tnf* and *Il23a* transcripts relative to *Actb* in neutrophils from the thymus and PB and cDCs from the B6 and NZW thymus were assessed by qRT–PCR. The means and SE of three independent experiments are shown. (C) The expression of the *Tnf* and *Il23a* transcripts relative to *Actb* in cultured neutrophils from the thymus and PB with or without stimulation were assessed by qRT–PCR. Data are representative of three independent experiments. The means and SE are shown. One-way ANOVA with Tukey *post hoc* test was performed (*P < 0.05, **P < 0.01, ***P < 0.001, NS = not significant).

Thymic neutrophils exhibit a higher expression level of CD14, an activation marker, than those in the PB of B6 mice and to a greater extent NZW mice (Fig. 4A). Therefore, we examined their cytokine expression under steady state conditions. Freshly isolated thymic neutrophils from NZW mice expressed larger amounts of TNFα (*Tnf*) mRNA compared with PB neutrophils, whereas *Tnf* mRNA levels in thymic neutrophils from B6 mice were much lower than in thymic neutrophils from NZW mice, but greater than those from PB neutrophils (Fig. 4B). It was reported that thymic cDCs produce IL-23 at basal level, but significantly up-regulated the production following irradiation (44). In our hands, *Il23a* mRNA was detected at a low level in thymic cDCs from both mouse strains (Fig. 4B) and F4/80^+^CD11b^+^ macrophages, B cells and pDCs from B6 mice, but were nearly undetectable in other thymic cells, including SP thymocytes, monocytes, under steady state conditions (Supplementary Figure 6). Notably, however, we found that thymic neutrophils from NZW mice and, to a lesser extent, those from B6 mice, exhibited high *Il23a* expression, although *Il23a* mRNA levels were undetectable in PB neutrophils from both mouse strains (Fig. 4B). We confirmed that both thymic and PB neutrophils from B6 mice showed increased *Il23a* and *Tnf* expression upon stimulation with mixture of Toll-like receptor ligands including CpG oligodeoxynucleotides, LPS and poly(I:C) *in vitro* (Fig. 4C). Thus, neutrophils associated with HCs in the thymus were constitutively activated and produced IL-23 under steady state conditions.

### Thymic pDCs are constitutively activated in an IL-23-dependent manner to produce IFNα

**Fig. 5.**
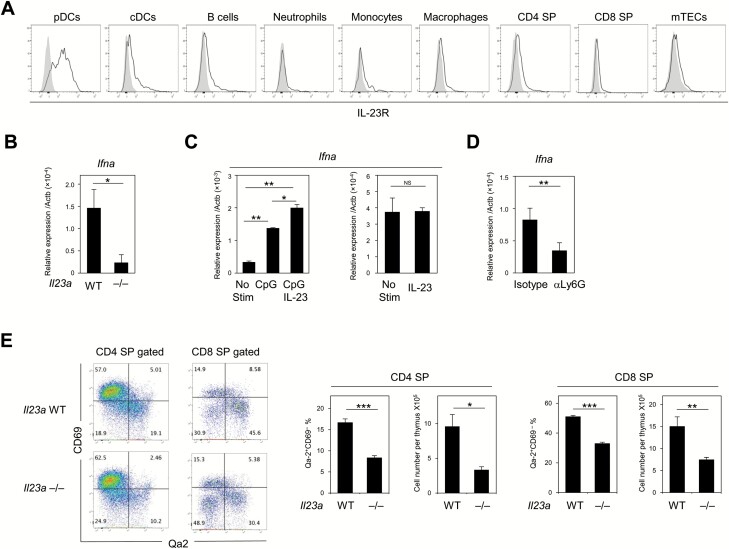
Thymic pDCs are constitutively activated in an IL-23-dependent manner. (A) IL-23R expression in the indicated cell types in the thymus of B6 mice (solid lines). Shaded regions indicate isotype control staining. Data are representative of three independent experiments. (B) The expression of the *Ifna* transcript relative to *Actb* in thymic pDCs from WT and *Il23a*^–/–^ mice was assessed by qRT–PCR. The means and SE of three independent experiments are shown. (C) The expression of the *Ifna* transcript relative to *Actb* were evaluated *in vitro* in stimulated B6 thymic pDCs with low-dose CpG (10 ng ml^−1^) plus IL-23 (100 ng ml^−1^) by qRT–PCR. Data are representative of three independent experiments. (D) Neutrophil depletion antibody (anti-Ly6G antibody) was injected as described in Methods, and the expression of the *Ifna* transcript relative to *Actb* in thymic pDCs was assessed by qRT–PCR. The means and SE of four independent experiments are shown. (E) CD69 and Qa2 expression in CD4 SP and CD8 SP thymocytes from WT and *Il23a*^–/–^ mice are shown (left). The means and SE of the percentages of the Qa2^+^CD69^−^ mature SP fraction in CD4^+^ and CD8^+^ SP thymocytes and their numbers from WT and *Il23a*^–/–^ mice of two independent experiments (*n* = 5) are shown (right). Unpaired student’s *t*-test (B, C right, D, E) and one-way ANOVA with Tukey *post hoc* test (C left) were performed (*P < 0.05, **P < 0.01, ***P < 0.001, NS = not significant).

To investigate the effects of thymic neutrophil activation associated with HC-mTECs, in particular via IL-23 production, we first examined the expression of IL-23 receptor (IL-23R) on thymic cells in B6 mice. We found that thymic pDCs (CD11c^low^ B220^+^PDCA-1^+^CCR9^+^) exhibited a strong expression of IL-23R; however, the expression was undetectable or only marginally detectable in mTECs, thymocytes, B cells and other myeloid cells (Fig. 5A and Supplementary Figure 7A). To investigate the involvement of IL-23 in thymic pDC activation, we examined *Il23a*^*–/–*^ mice. The number and proportion of thymic neutrophils in *Il23a*^*–/–*^ mice tended to decrease compared with those observed in wild-type (WT) mice, whereas the number and proportion of thymic pDCs increased (Supplementary Figure 7B and C). MHC II expression in thymic pDCs, cDCs and B cells was comparable between WT and *Il23a*^*–/–*^ mice (Supplementary Figure 7D). Notably, however, thymic pDCs in *Il23a*^*–/–*^ mice exhibited significantly less expression of *Ifna* compared with WT mice (Fig. 5B), suggesting the involvement of IL-23 in thymic pDC activation. pDCs exhibited significantly higher expression of *Ifna* transcripts than all other cell types in the thymus (Supplementary Figure 7E), suggesting that pDCs are the major source of IFNα in the steady state thymus. Furthermore, the addition of IL-23 to sorted pDC cultures enhanced low-dose CpG-mediated *Ifna* expression, and IL-23 alone failed to induce *Ifna* expression (Fig. 5C). Importantly, neutrophil depletion by 2-week anti-Ly6G antibody injection resulted in a reduction of *Ifna* expression in thymic pDCs (Fig. 5D), indicating a role of neutrophils on pDC activation.

Type I interferons play a role in SP thymocyte maturation (13). Thus, SP cells expressing Qa2, a marker of type I interferon exposure (13), and of SP thymocyte maturation (45), were significantly decreased in *Ifnar2*^*–/–*^ mice, in which IFNα-induced signaling is canceled (46), a feature also seen in *Ifnar1*^*–/–*^ mice (13) (Supplementary Figure 8). Consistently, Qa2^+^ SP cells were significantly decreased in *Il23a*^*–/–*^ mice, although overall thymocyte development was apparently unaffected (Fig. 5E and Supplementary Figure 9). These results suggested that thymic pDCs expressed IL-23R and were constitutively activated by neutrophils to produce IFNα for promoting the maturation of SP thymocytes. Thus, IL-23 produced from neutrophils activated by HCs may amplify the spontaneous and basal level of pDC activation, strongly suggesting an HC-neutrophil-pDC cellular axis in the thymic medulla.

### Aire deficiency compromises the HC-neutrophil-pDC axis

**Fig. 6.**
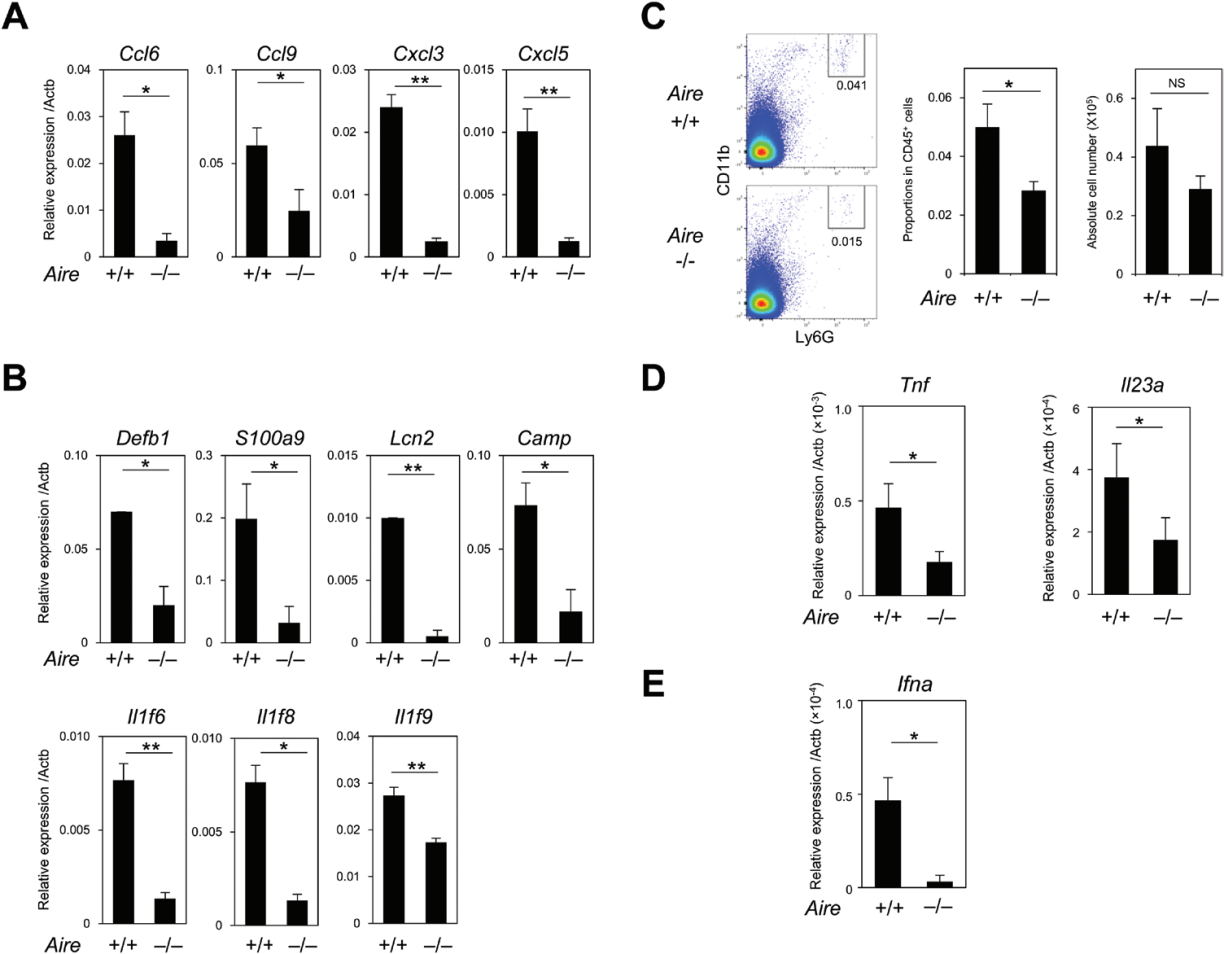
Thymic neutrophil recruitment and pDC activation are impaired in *Aire*^*–/–*^ mice. (A, B) The expression of the transcripts of the indicated genes relative to *Actb* in freshly isolated mTECs from Aire^+/+^ and Aire^–/–^ mice was assessed by qRT–PCR. The means and SE of three independent experiments are shown. (C) Percentages of thymic CD11b^+^Ly6G^+^ neutrophils in CD45^+^ cells from Aire^+/+^ and Aire^–/–^ mice are indicated (left). The means and SE of the percentages and cell numbers of thymic neutrophils from Aire^+/+^ and Aire^–/–^ mice from four independent experiments were shown (right). (D) The expression of the *Tnf* and *Il23a* transcripts relative to *Actb* in thymic neutrophils from Aire^+/+^ and Aire^–/–^ mice was assessed by qRT–PCR. (E) The expression of the *Ifna* transcript relative to *Actb* in thymic pDCs was assessed by qRT–PCR. The means and SE of three independent experiments are shown. Unpaired student’s *t*-test was performed (*P < 0.05, **P < 0.01, NS = not significant).

Because *Aire*^*–/–*^ mice poorly develop HC-mTECs (20) and have defects in SP thymocyte maturation (45), we investigated the thymus of *Aire*^*–/–*^ mice to define the role of HC-mTECs in the constitutive activation of thymic neutrophils and pDCs. Sorted mTECs from *Aire*^*–/–*^ mice showed markedly reduced gene expression of chemokines (*Ccl6*, *Ccl9*, *Cxcl3* and *Cxcl5*), IL-36-family cytokines (*Il1f6*, *Il1f8* and *Il1f9*) and antimicrobial peptides (*Defb1*, *S100a9*, *Lcn2* and *Camp*) compared with control mice (Fig. 6A and B). These reductions correlated with the decrease in HC-mTECs. In thymus tissue from *Aire*^*–/–*^ mice, the proportion of neutrophils decreased significantly (Fig. 6C and Supplementary Figure 10), and sorted thymic neutrophils from *Aire*^*–/–*^ mice showed reduced expression of *Il23a* and *Tnf* as compared with control mice (Fig. 6D). We also found that pDCs from the thymus of *Aire*^*–/–*^ mice showed a significant decrease in *Ifna* expression compared with *Aire*^*+/+*^ mice (Fig. 6E), but their proportion and number increased (Supplementary Figure 11A). Furthermore, MHC II expression in thymic pDCs and cDCs from *Aire*^*–/–*^ mice was unaffected, whereas thymic B cells showed slightly reduced MHC II expression as previously reported (Supplementary Figure 11B) (8). Because thymic pDCs express no detectable levels of Aire (8), the decrease in *Ifna* expression in pDCs from *Aire*^*–/–*^ mice was not attributable to the direct effects of *Aire* deficiency. These results strongly suggested that an Aire-dependent development of HC-mTECs played an important role in the spontaneous activation of thymic neutrophils and pDCs (Supplementary Figure 12).

## Discussion

HCs are unique epithelial structures specifically detected in the thymic medulla, but their significance and function have remained enigmatic. HCs often show evidence of cornification reminiscent of the epidermis, with an abundant expression of molecules related to keratinocytes, such as K1, K10, involucrin and Dsg3 (16–20). Additionally, HC-mTECs strongly express Cld3/4, a marker of Aire^+^ mTECs (35), and thus HCs represent a terminally differentiated Aire-expressing mTEC lineage (21–23). In this study, we found that SASPase, which is expressed during the cornification process of the epidermis (29, 30), is also highly and specifically expressed in HC-like mTEC clusters by immunohistochemistry. Taking advantage, we sorted a rare HC-mTECs population, defined as the 2% of mTECs with the highest EGFP expression from SASP-EGFP-KI mice. The SASP^high^ mTEC fraction showed higher expression of HC-mTEC markers including *Krt1*, *Krt10* and *Ivl* and lower Aire expression compared to the SASP^neg/low^ mTEC fractions, suggesting highly mature mTECs. On the other hand, previous lineage-tracing experiments revealed that post-Aire, terminally mature mTECs down-regulate MHC II and CD80 and are characterized as HC-mTECs, although the locations of post-Aire mTECs in the medulla are not well characterized (20–23). Indeed, the SASP^high^ mTEC fraction contained a minor (~30%) MHC II^low^ fraction that exhibited the highest *Krt10* and lowest *Aire* expression among mTEC fractions. Using immunohistochemistry, we found most of the K10^+^ cells were MHC II^low/neg^, as previously reported (17). Thus, the low proportion of the MHC II^low^ fraction from the flow cytometry experiments may be due to the loss of dying HC-mTECs during single-cell preparation. These results strongly suggest that SASP^high^ mTECs are highly matured mTECs that are undergoing clustering, cornifying and differentiating into HC-mTECs, and MHC II expression is down-regulated during their terminal maturation of SASP^high^ mTECs. Thus, SASP-EGFP-KI mice are a useful tool to identify HC-mTECs, based on the histological criteria originally described by Dr Arthur Hill Hassall (15).

We demonstrated that SASP^high^ HC-mTECs exhibited a high expression of genes potentially related to inflammation, including chemokines (*Ccl6*, *Ccl9*, *Cxcl3* and *Cxcl5*), pro-inflammatory cytokines (*Il1f6*, *Il1f8* and *Il1f9*) and antimicrobial peptides (*Defb1*, *S100a9* and *Lcn2*), compared to other mTECs. It is unlikely that the observed gene expression represented classical TSA gene expression under the direct control of Aire (47), because the majority of mature mTECs expressing *Aire* barely expressed these genes. Furthermore, CXCL5 protein was clearly detected in almost all HC-mTECs by immunostaining, unlike typical Aire-dependent TSAs, which are expressed in a rare proportion of mTECs (47). Notably, we also found that HC-mTECs showed a marked increase in *Cdkn2a* and *Cdkn1a* accompanied by significant SA-β-Gal expression, suggestive of cellular senescence (43). Therefore, a more likely explanation is that the increase in inflammatory gene expression observed in HC-mTECs represents a senescence-associated secretory phenotype (42). It might be possible that double-stranded DNA breaks promoted by Aire (47–50) and constitutive NF-κB activation during mTEC maturation underlie cell senescence and the senescence-associated secretory phenotype (51). Alternatively, our findings could reflect cellular stress induced in terminally differentiated, cornified mTECs, which is unlike terminally differentiated keratinocytes in the epidermis because of the lack of open space necessary to peel off. Consequently, thymic HCs might provide a unique example of senescent cells playing a physiological role in tissue.

In agreement with the strong expression of CXCL5 in HC-mTECs, neutrophils expressing CXCR2 are mostly localized in medullary regions, especially in NZW mice. Typically, NZW mice showed multiple large clusters of mTECs, which integrated aggregated neutrophils into structures, like those observed in the HCs of human thymus. Freshly isolated thymic neutrophils showed marked increases in CD14 and *Tnf* expression compared with PB neutrophils, and most notably exhibited elevated *Il23a* expression, which was undetectable in PB neutrophils, indicating that thymic neutrophils had been constitutively activated *in situ*. IL-23 is produced by DCs under various pathogenic conditions involving T_h_17 cell activation (52), and in the thymus, IL-23 is produced by CD103^+^ cDCs following γ-ray irradiation to stimulate lymphoid tissue inducer cells and promote TEC recovery (44). However, under steady state conditions, we found that thymic neutrophils were the major population expressing *Il23a* in the thymus, although the ability of *Il23a* expression was not unique to thymic neutrophils, because PB neutrophils similarly showed increased *Il23a* expression upon activation *in vitro*. Concomitant with thymic neutrophil activation, we also found that thymic pDCs showed evidence of activation, especially *Ifna* expression. Additionally, NZW mice, which exhibit increased HC-mTECs, showed a higher activation status of thymic neutrophils and pDCs compared to B6 mice. Because pDCs are essentially the only thymic cell population expressing IL-23R at high levels, it appears reasonable to assume that thymic pDC activation was mediated via IL-23 derived from neutrophils associated with HC-mTECs. Supporting this assumption, the spontaneous activation of thymic pDCs to express *Ifna* was significantly compromised in *Il23a*^*–/–*^ mice.

pDCs are activated by a variety of damage-associated molecular patterns (DAMPs). HC-mTECs are intimately associated with activated neutrophils, suggesting these activated neutrophils could be a source of DAMPs in the thymic medulla necessary for pDC activation as previously reported (53). Furthermore, IL-1-family cytokines as well as antimicrobial peptides highly expressed in HC-mTECs might also contribute to thymic pDC activation. We observed no evidence of direct thymic pDC activation by IL-23, at least *in vitro*, although treatment with optimal doses of CpG significantly initiated pDC activation. Notably, however, IL-23 significantly enhanced thymic pDC activation in the presence of low doses of CpG, suggesting that IL-23 functions as part of an amplification pathway associated with physiological levels of DAMP-mediated pDC activation in the thymus. These results collectively suggested that HC-mTECs sustained the endogenous activation of pDCs to express *Ifna* in the medulla through constitutive neutrophil activation, including a continuous and effective supply of DAMPs and IL-23.

Aire plays an important role throughout mTEC differentiation and maturation beyond the control of TSA expression, and accordingly, *Aire*^*–/–*^ mice show compromised development of HCs (20). We confirmed that characteristic gene expression in HC-mTECs was markedly decreased in the mTECs of *Aire*^*–/–*^ mice. Concordantly, the spontaneous activation of both neutrophils and pDCs in the thymus was markedly compromised in *Aire*^*–/–*^ mice, reinforcing HC-mTECs as crucial for the constitutive activation of thymic neutrophils and pDCs. Recent reports revealed that human autoimmune polyendocrinopathy-candidiasis-ectodermal dystrophy patients exhibiting *Aire* deficiency often develop high-affinity auto-antibodies against various inflammatory cytokines, including type I interferons, IL-17, IL-22 and defensins, possibly contributing to the amelioration of type I diabetes and increased susceptibility to mucocutaneous candidiasis and gastrointestinal autoimmunity (9, 54–56). Thus, there is the intriguing possibility that the decreased expression of these pro-inflammatory cytokines in *Aire*-deficient thymus may also contribute to the impaired T-cell self-tolerance against these factors.

The thymus, particularly in the medulla, shows relatively high levels of type I interferons under steady state conditions in both mice and humans compared to other organs (9–12). Tonic interferon signaling plays a role in the expression of Qa2 as well as STAT1 and IRF7 in SP thymocytes, licensing the cells to respond to cytokines found in the periphery (13). However, the expression of type I interferons during infection, which could cause thymic involution (57), might be much higher compared to that at the steady state. Our results suggested that thymic pDCs under the influence of HC-mTECs might represent one of the sources of IFNα in steady state conditions. Consistently, SP thymocytes in *Il23a*^*–/–*^ and *Aire*^*–/–*^ mice showed fewer Qa-2^+^ mature SP thymocytes. These findings might also explain the mechanisms associated with the delayed maturation and export of SP thymocytes in *Aire*^*–/–*^ mice (45, 58–60).

NZW mice displayed prominent HCs along with increased activation of both thymic neutrophils and pDCs compared with other mouse strains. NZW mice strongly promote the development of overt lupus disease when crossed with lupus-prone New Zealand Black (NZB) mice, although NZW mice *per se* rarely develop lupus disease, probably because of the absence of genetic B-cell abnormality (61). Based on this observation, it is notable that untreated SLE patients and those with autoimmune MG with thymic hyperplasia show large and complex HCs and increased numbers of HCs, respectively, in the thymus (25, 62). Additionally, T cells in SLE patients show characteristic gene signatures related to type I interferon signaling and granulopoiesis (63). These results might imply that constitutive but low levels of IFNα induced by HCs may contribute to the physiological thymic function, whereas HC hyperplasia and a subsequent increase of IFNα production in the thymic medulla might be related to certain types of systemic autoimmunity, although further studies are needed.

In conclusion, we demonstrated that HCs developed from mTECs under the control of Aire showed features of cell senescence and spontaneously initiated the constitutive activation of thymic neutrophils and pDCs to generate a sterile inflammatory environment in the thymic medulla that promotes IFNα production. This IFNα production in turn may promote SP thymocyte maturation. Our results demonstrate a novel functional mechanism associated with Aire through HC formation in the establishment of the thymic microenvironment.

## Funding

This work was supported by grants from the Japanese Ministry of Education, Culture, Science, Sports, and Technology (24590580, 15H01154, 17H05641, 18H02640, 18K19442 to Y.H.; 24111008 to N.M); Japan Agency for Medical Research and Development under Grant Number (JP18gm5010001) to Y.H.; iPS Cell Research Fund to Y.H.; and the Takeda Science Foundation to Y.H.

*Conflicts of interest statement:* The authors declared no conflicts of interest.

## Supplementary Material

dxy073_suppl_Supplementary_Figures

dxy073_suppl_Supplementary_Table_S1

dxy073_Suppl_Supplementary_Figure_Legends
