# Supplementary material for: Hassall’s corpuscles with cellular-senescence features maintain IFNα production through neutrophils and pDC activation in the thymus
Source: Int Immunol. 2018 Dec 10;31(3):127–39. doi: 10.1093/intimm/dxy073 (PMC9271218; doi:10.1093/intimm/dxy073)
Supplement: dxy073_suppl_Supplementary_Figures [file dxy073_suppl_supplementary_figures.pdf]

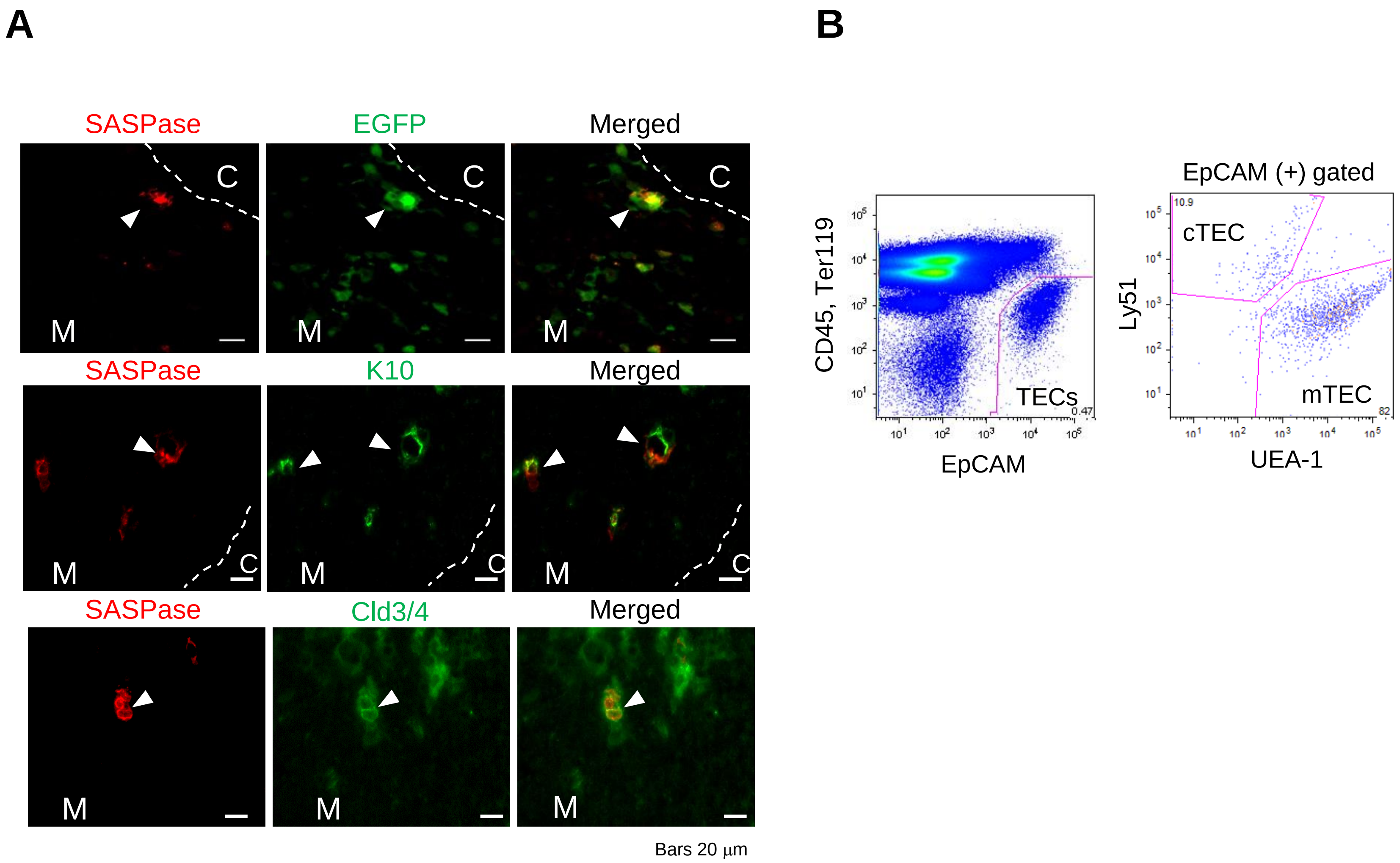

Supplemental Figure 1

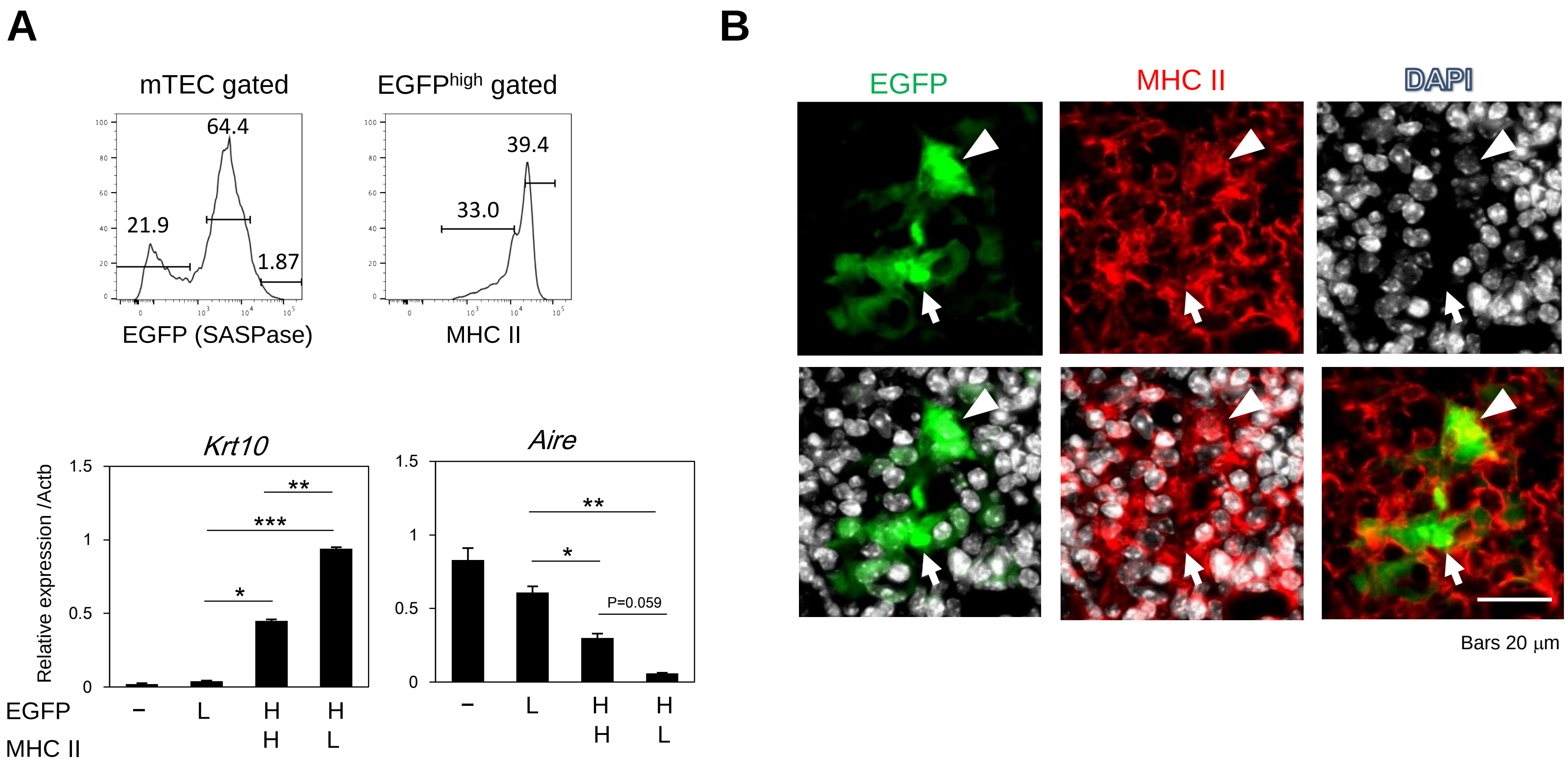

Supplemental Figure 2

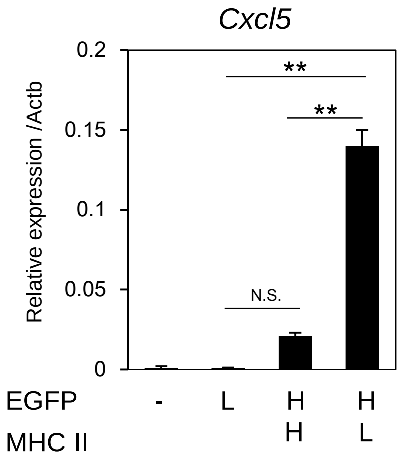

Supplemental
Figure 3

A

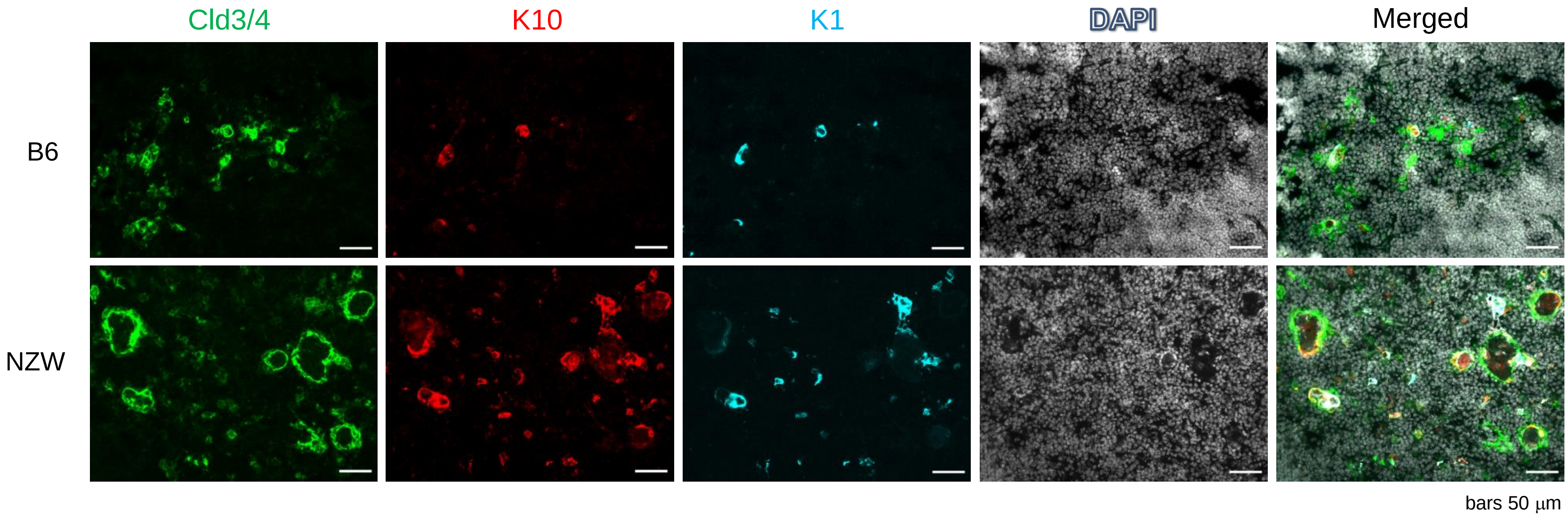

B

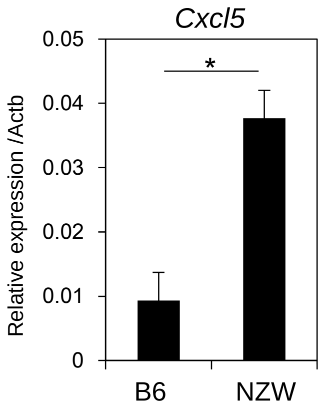

Supplemental Figure 4

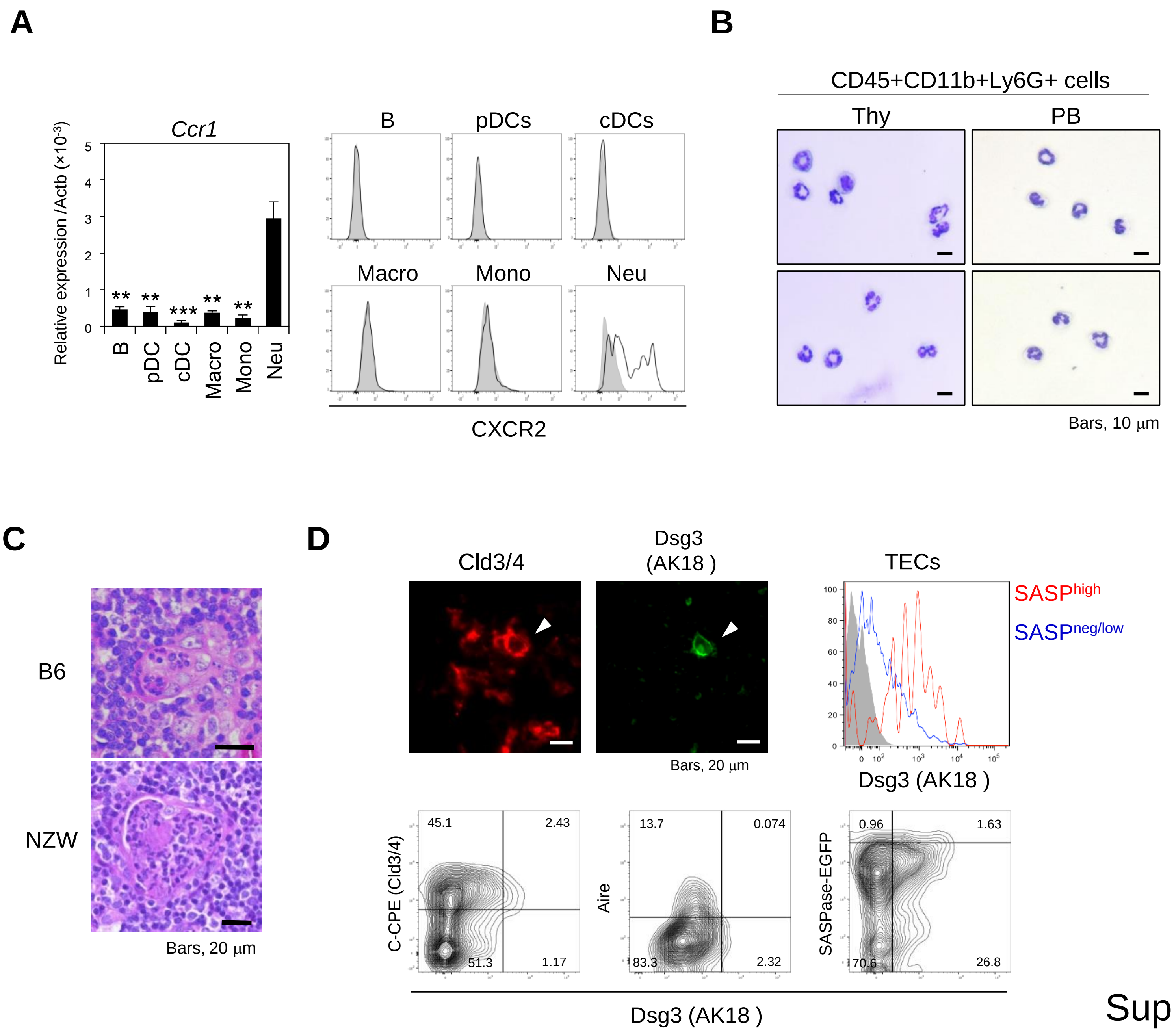

Supplemental Figure 5

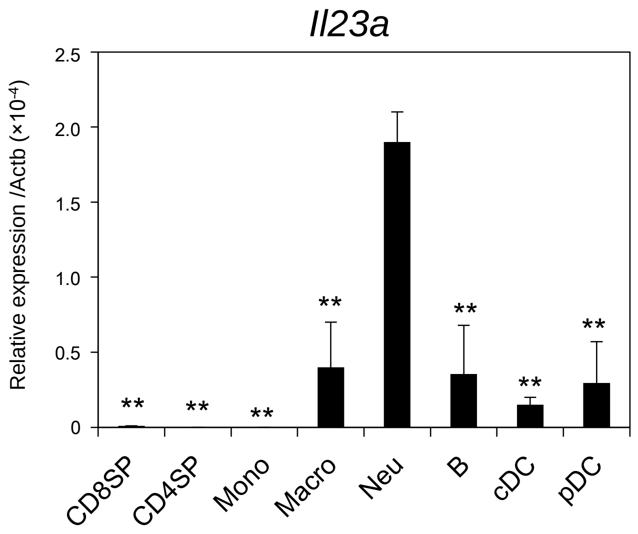

Supplemental
Figure 6

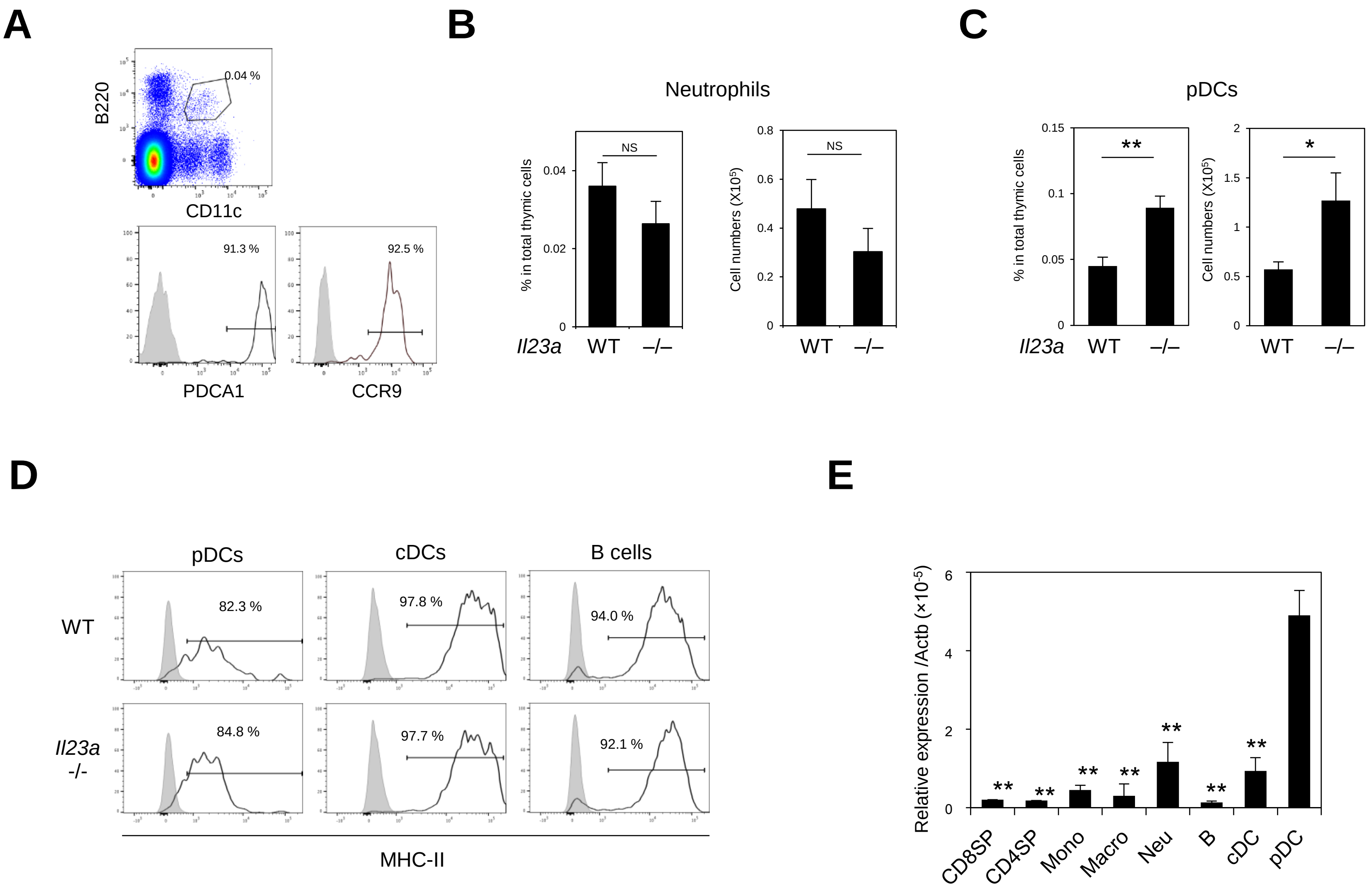

Supplemental Figure 7

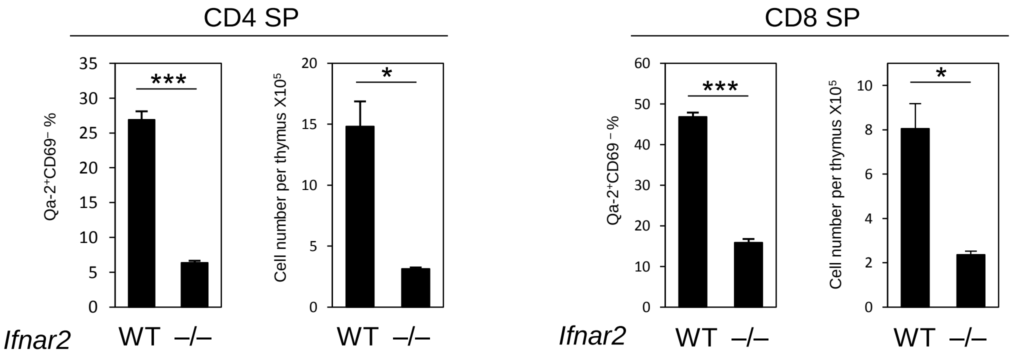

Supplemental Figure 8

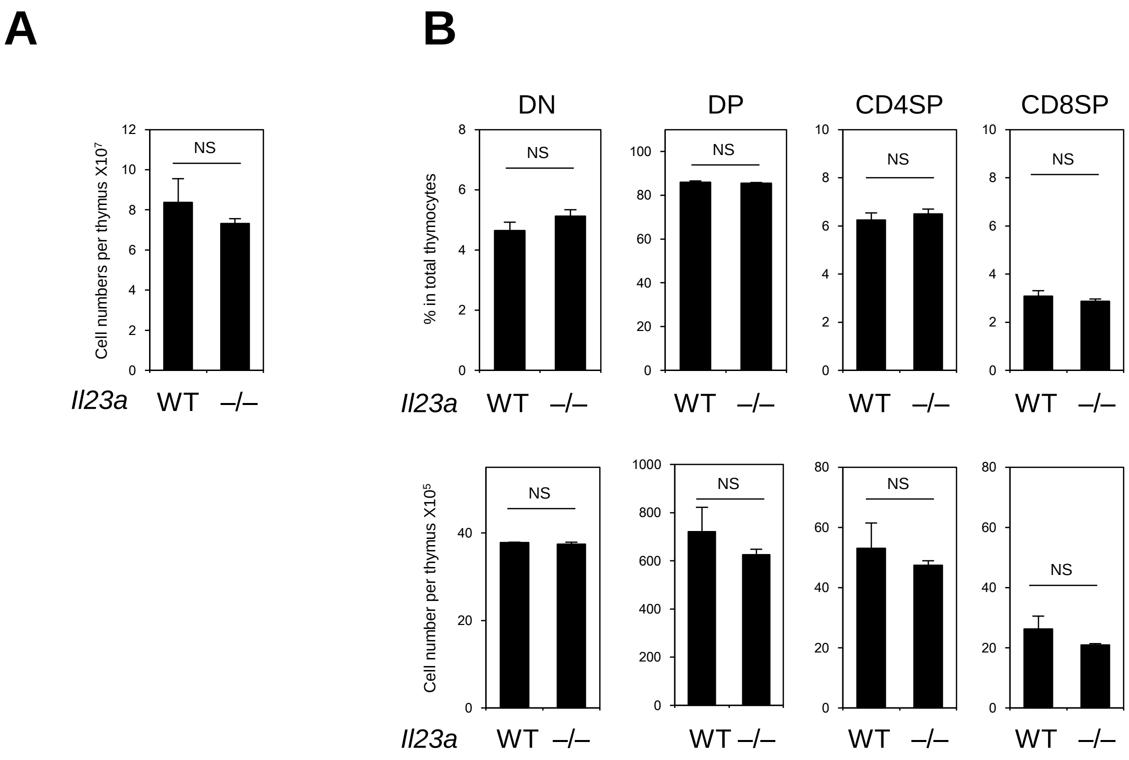

Supplemental Figure 9

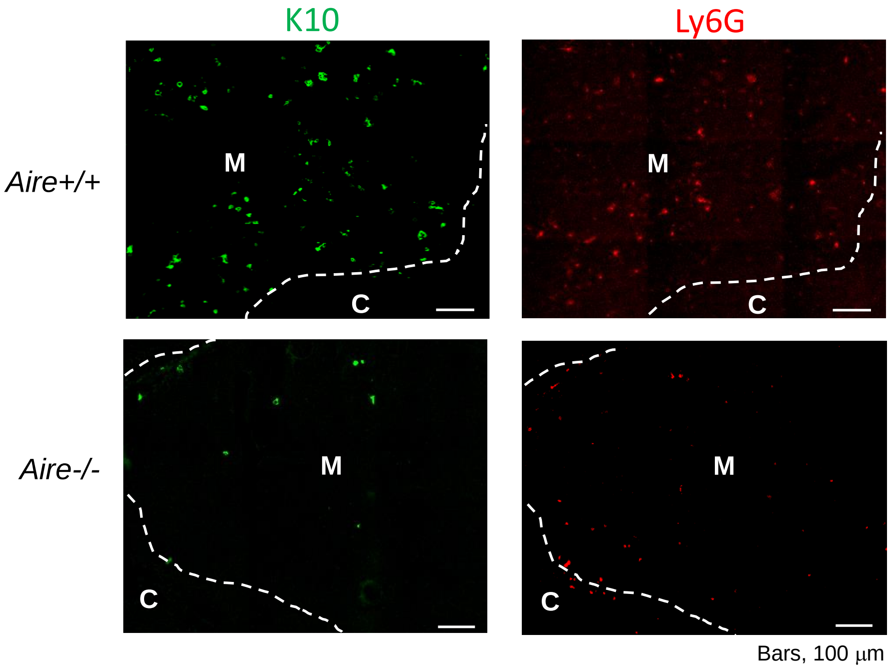

Supplemental Figure 10

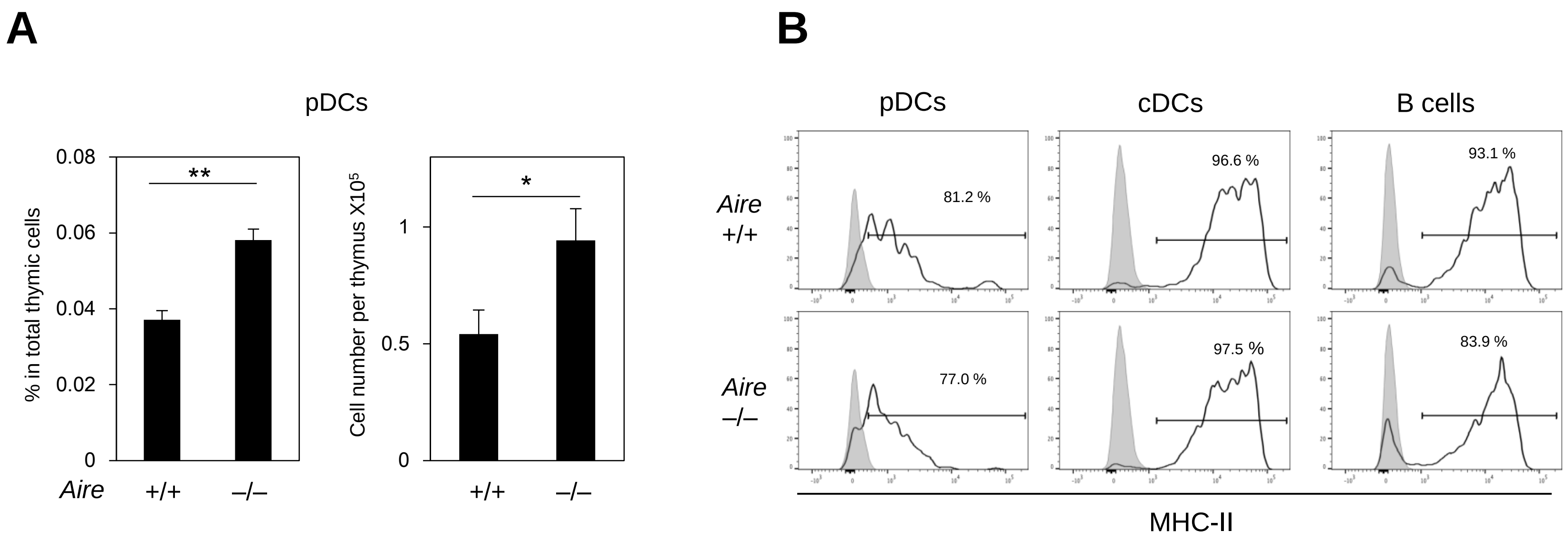

Supplemental Figure 11

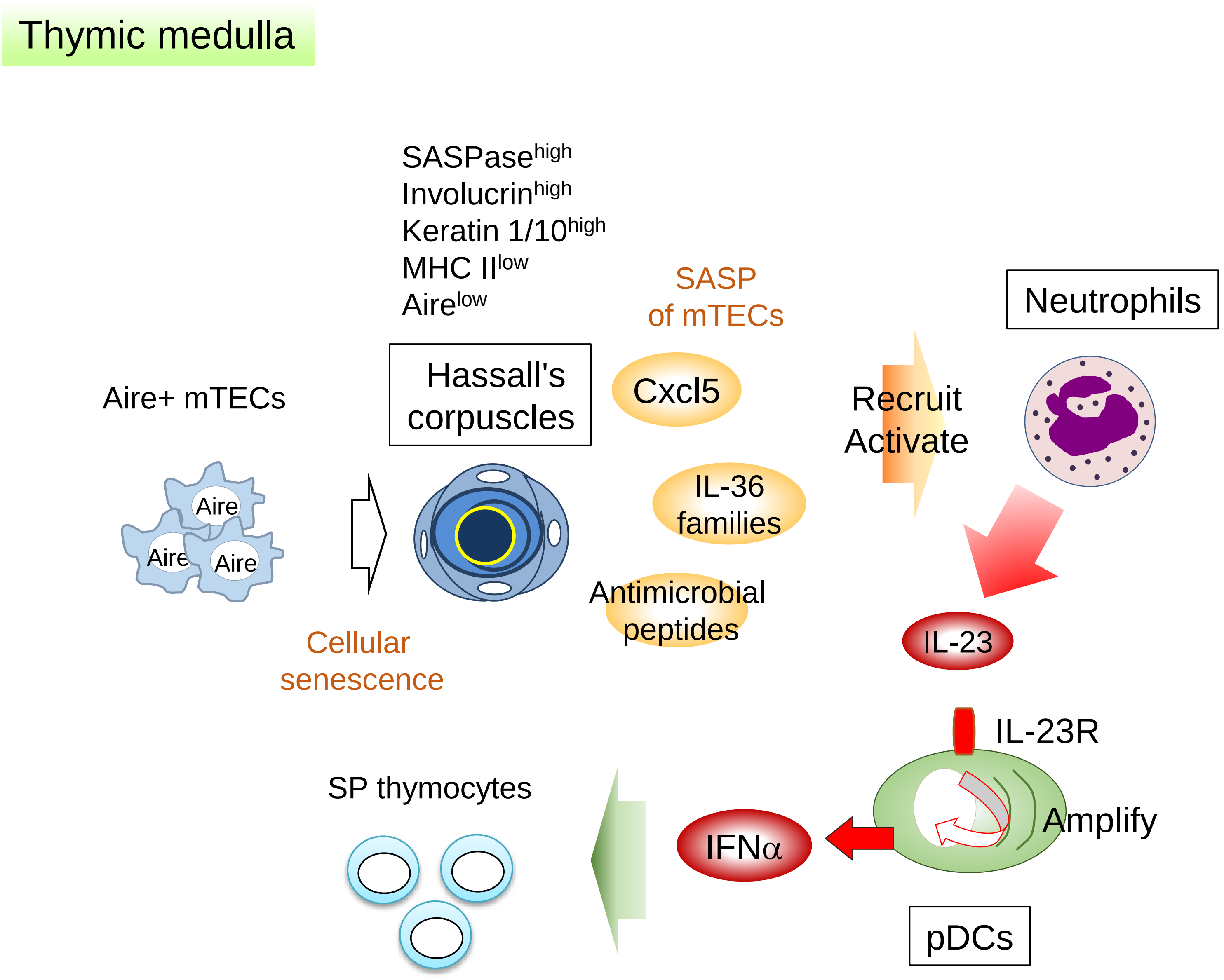

Supplemental Figure 12
