# Supplementary material for: Hassall’s corpuscles with cellular-senescence features maintain IFNα production through neutrophils and pDC activation in the thymus
Source: Int Immunol. 2018 Dec 10;31(3):127–39. doi: 10.1093/intimm/dxy073 (PMC9271218; doi:10.1093/intimm/dxy073)
Supplement: dxy073_suppl_Supplementary_Table_S1 [file dxy073_suppl_supplementary_table_s1.docx]

| Gene name | Forward primer 5’-3’ | Reverse primer 5’-3’ |
| --- | --- | --- |
| *Actb* | AAGGCCAACCGTGAAAAGAT | GTGGTACGACCAGAGGCATAC |
| *Aire* | TCCTCAATGAGCACTCATTTGAC | CCACCTGTCATCAGGAAGAG |
| *Asprv1* | GGTAGGGGGACTTTGTGTTG | TGGCTAGGCCGGATGATAC |
| *Ccl6* | CTCCAAGACTGCCATTTCATT | GCGACGATCTTCTTTTTCCA |
| *Ccl9* | TGGGCCCAGATCACACAT | CCCATGTGAAACATTTCAATTTC |
| *Ccl17* | TGCTTCTGGGGACTTTTCTG | GAATGGCCCCTTTGAAGTAA |
| *Ccl19* | TGTGGCCTGCCTCAGATTAT | AGTCTTCCGCATCATTAGCAC |
| *Ccl21* | TCCAAGGGCTGCAAGAGA | TGAAGTTCGTGGGGGATCT |
| *Ccl22* | TCTTGCTGTGGCAATTCAGA | GAGGGTGACGGATGTAGTCC |
| *Ccr1* | TGGACAAAATACTCTGGAAACACA | TGTGAAATCTGAAATCTCCATCC |
| *Cd40* | AAGGAACGAGTCAGACTAATGTCA | AGAAACACCCCGAAAATGGT |
| *Cdkn1a* | CAGATCCACAGCGATATCCA | GGCACACTTTGCTCCTGTG |
| *Cdkn2a* | GGGTTTTCTTGGTGAAGTTCG | TTGCCCATCATCATCACCT |
| *Cdkn2d* | ACACCTGTCCATTGAAGAAGG | CCCCAAACACACACACTCAA |
| *Cxcl3* | CCCCAGGCTTCAGATAATCA | TCTGATTTAGAATGCAGGTCCTT |
| *Cxcl5* | GGGAAACCATTGTCCCTGA | TCCGATAGTGTGACAGATAGGAAA |
| *Defb1* | GGCTGCCACCACTATGAAA | TGTGAGAATGCCAACACCTG |
| *Foxn1* | TGACGGAGCACTTCCCTTAC | GACAGGTTATGGCGAACAGAA |
| *Ifna* | ACCCAGCAGATCCTGAACAT | AATGAGTCTAGGAGGGTTGTATTCC |
| *Il1f6* | TGTGCAGACACATTCCTATTCA | CGAAGGTGATGCTGCTCTTAG |
| *Il1f8* | CTTCGATCCCAGAGACAAGACT | ATTCGGTTCCCACATTTGAA |
| *Il1f9* | GGGTTCTGGCACTCTCTCC | CGGCAAAGCTTTATTGCTTAC |
| *Il7* | CTGCTGCAGTCCCAGTCAT | TCAGTGGAGGAATTCCAAAGAT |
| *Il23a* | TCCCTACTAGGACTCAGCCAAC | AGAACTCAGGCTGGGCATC |
| *Ins2* | GAAGTGGAGGACCCACAAGT | AGTGCCAAGGTCTGAAGGTC |
| *Ivl* | GTGAGTTTGTTTGGTCTACAG | GAAAGCCCTTCTCTTGAATCTC |
| *Krt1* | TTTGCCTCCTTCATCGACA | GTTTTGGGTCCGGGTTGT |
| *Krt10* | GTTCAATCAGAAGAGCAAGGA | GTAGTTCAATCTCCAGACCC |
| *Lcn2* | ATGTCACCTCCATCCTGGTC | CCTGTGCATATTTCCCAGAGT |
| *Ltbr* | GCTCCAGGTACCTCCTACTCG | ATGGCCAGCAGTAGCATTG |
| *Mx1* | TTCAAGGATCACTCATACTTCAGC | GGGAGGTGAGCTCCTCAGT |
| *MhcII* | CTCAGAAATAGCAAGTCAGTC | AATCTCAGGTTCCCAGTG |
| *Tslp* | GACAGCATGGTTCTTCTCAG | CTGGAGATTGCATGAAGG |
| *S100a9* | GACACCCTGACACCCTGAG | TGAGGGCTTCATTTCTCTTCTC |
| *Tnf* | TCTTCTCATTCCTGCTTGTGG | GGTCTGGGCCATAGAACTGA |
| *Tnfrsf11a* | GTGCTGCTCGTTCCACTG | AGATGCTCATAATGCCTCTCCT |
| *Xcr1* | ATTACCTTTGGGGCAGTTTAGA | TGGTTGAAAGAACAGGATCTGA |
