# Supplementary material for: Hassall’s corpuscles with cellular-senescence features maintain IFNα production through neutrophils and pDC activation in the thymus
Source: Int Immunol. 2018 Dec 10;31(3):127–39. doi: 10.1093/intimm/dxy073 (PMC9271218; doi:10.1093/intimm/dxy073)
Supplement: dxy073_Suppl_Supplementary_Figure_Legends [file dxy073_suppl_supplementary_figure_legends.docx]

**Figure S1. SASPase proteins co-localize with EGFP and mTEC markers in the thymic medulla.** (A) The thymus from SASP-EGFP-KI mouse was two-color stained with anti-SASPase polyclonal antibody and rat anti-EGFP antibody (upper). B6 mouse thymi were two-color stained using antibodies against SASPase (clone mG2-C) and K10 (middle) or Cld3/4 (lower). Arrowheads indicate HC-like structures. White broken lines indicate the cortico-medullary borders. Scale bars, 20 μm. C, cortex; M, medulla. (B) Representative FACS profiles using indicated markers to gate the cTEC and mTEC fractions are shown. Data are representative of two (A) and three (B) independent experiments.

**Figure S2. Both MHC II^high^ and MHC II^low^ fractions in EGFP^high^ mTECs show characteristics of HC-mTECs.** (A) Representative FACS profiles using the indicated markers to gate MHC II^low^ and MHC II^high^ cells in the EGFP^high^ fraction (upper). The expressions of the *Krt10* and *Aire* transcripts relative to Actb in the indicated cell fractions from SASP-EGFP-KI mice were assessed by qPCR. Representative of two independent experiments (means and SE) are shown. One-way ANOVA with Tukey post-hoc test was performed (*p<0.05, **p<0.01, ***p<0.001) (lower). L, low; H, high. (B) The thymus from an SASP-EGFP-KI mouse was multi-color stained with the indicated antibodies. MHC II^low^ (arrow) and MHC II^high^ (arrowhead) cells in EGFP^high^ cells are indicated. Data are representative of at least three independent experiments.

**Figure S3. The MHC II^low^ fraction expresses higher *Cxcl5* expression compared to the MHC II^high^ fraction in SASP^high^ mTECs.** The expression of the *Cxcl5* transcript relative to Actb in the indicated cell fractions from SASP-EGFP-KI mice was assessed by qPCR. L, low; H, high. Representative of two independent experiments (means and SE) is shown. One-way ANOVA with Tukey post-hoc test was performed (NS = not significant, **p<0.01)

**Figure S4.**  **NZW mice harbor higher number of and larger HC-like structures and elevated *Cxcl5* expression relative to B6 mice.** (A) Thymus tissues from adult B6 and NZW mice were multi-color immunostained using the indicated antibodies and reagents: Cld3/4, Keratin 10 (K10), Keratin 1 (K1), and 4,6-diamidino-2-phenylindole (white). HCs were detected as Cld3/4^+^ areas. Scale bars, 50 μm. (B) The expression of the *Cxcl5* transcript relative to [*Actb*](https://www.ncbi.nlm.nih.gov/gene/11461) in total mTECs from B6 and NZW mice was assessed by qPCR. The means and SE of three independent experiments are shown. Unpaired student’s t test was performed (*p<0.05).

**Figure S5.**  ***Ccr1* and CXCR2 are preferentially expressed in thymic neutrophils.** (A) : The expression of the *Ccr1* transcript relative to [*Actb*](https://www.ncbi.nlm.nih.gov/gene/11461) in sorted B cells (B), pDCs (pDC), cDCs (cDC), macrophages (Macro), monocytes (Mono), and neutrophils (Neu) from the thymus of B6 mice was assessed by qPCR. The means and SE of two independent experiments are shown. Statistical analysis compared to Neu are shown. One-way ANOVA with Tukey post-hoc test was performed (**p<0.01, ***p<0.001) (left). CXCR2 expression in each cell fraction was examined by flow cytometry (solid line). Shaded regions indicate isotype-control staining (right). Data are representative of three independent experiments. (B) CD11b^+^Ly6G^+^ cells from the thymus and PB of B6 mice were sorted and cytospinned. Giemsa staining images are shown. Scale bars, 10 μm. Data are representative of three independent experiments. (C) Thymus tissues from both B6 and NZW mice stained with hematoxylin and eosin. Scale bars, 20 μm. Data are representative of three independent experiments. (D) Thymus tissues from B6 mice stained with antibodies against Cld3/4 and Dsg3 (left). Dsg3 expression in the SASP^neg/low^ (blue line) and SASP^high^ (red line) fractions of TECs from SASP-EGFP-KI mice analyzed by flow cytometry with the same gating strategy as Supplemental Figure 1B. Shaded regions indicate isotype-control staining (right upper). Concurrent Dsg3 expression with C-CPE (left lower), Aire (middle lower), and SASP-EGFP (right lower) in TEC gates are shown. HC-mTECs shown in Fig. 3D were identified as Cld3/4^+^Dsg3^+^ mTECs. Data are representative of three independent experiments.

**Figure S6. *Il23a* is preferentially expressed in thymic neutrophils*.*** The expression of the *Il23a* transcript relative to [*Actb*](https://www.ncbi.nlm.nih.gov/gene/11461) in sorted CD8 single positive thymocytes (CD8 SP), CD4 single positive thymocytes (CD4 SP), monocytes (Mono), macrophages (Macro), neutrophils (Neu), B cells (B), cDCs (cDC), and pDCs (pDC) from the thymus of B6 mice was assessed by qRT-PCR. The means and SE of two independent experiments are shown. Statistical analysis compared to Neu was shown. One-way ANOVA with Tukey post-hoc test was performed (**p<0.01).

**Figure S7. Thymic B cells and cDCs are unaffected in *Il23a***^–/–^ **mice.** (A) PDCA-1 and CCR9 expressions in the CD11c^lo^ B220^+^ thymic pDC fraction were examined by flow cytometry. (B) Percentages and cell numbers of thymic neutrophils in WT and *Il23a*^–/–^ mice (*n* = 5). Data are shown as the means and SE. (C) Percentages and cell numbers of thymic pDCs in WT and *Il23a*^–/–^ mice (*n* = 5). Data are shown as the means and SE. (D) MHC II expressions in thymic pDC (CD11c^lo^ B220^+^), cDCs (CD11c^high^ B220^-^), and B cells (CD11c^–^ B220^+^) were examined by flow cytometry, with the MHC-II-positive percentages shown. Unpaired student’s t test was performed (*p<0.05, **p<0.01, NS = not significant). Data are representative of at least three independent experiments (A-D). (E) The expression of the *Ifna* transcript relative to [*Actb*](https://www.ncbi.nlm.nih.gov/gene/11461) in sorted CD8 single positive thymocytes (CD8 SP), CD4 single positive thymocytes (CD4 SP), monocytes (Mono), macrophages (Macro), neutrophils (Neu), B cells (B), cDCs (cDC), and pDCs (pDC) from the thymus of B6 mice assessed by qRT-PCR. The means and SE of two independent experiments are shown. Statistical analysis compared to pDC was shown. One-way ANOVA with Tukey post-hoc test was performed (**p<0.01).

**Figure S8. Single positive thymocyte maturation is impaired in *Ifnar2*^–/–^ mice.** Percentages and numbers of the Qa2^+^CD69^−^ fraction in CD4 and CD8 SP thymocytes from WT and *Ifnar2*^–/–^ mice from two independent experiments (n = 6). Data are shown as the mean and SE. Unpaired student’s t test was performed (*p<0.05, ***p<0.001).

**Figure S9.** **Total thymocyte numbers and subsets are unaffected in *Il23a***^–/–^ **mice.** Total thymocyte number (left) and the number and proportion of thymocyte subsets [CD4/8 double negative (DN), CD4/8 double positive (DP), CD4 SP, and CD8 SP] (right) from WT and *Il23a*^–/–^ mice from two independent experiments (*n* = 10). Data are shown as the mean and SE. Unpaired student’s t test was performed (NS = not significant).

**Figure S10.** **Thymic neutrophils and K10**^+^ **mTECs decrease in *Aire*^–/–^ mice.**

Thymus tissues from adult *Aire*^+/+^ and *Aire*^–/–^ mice were two-color immuno-stained using the indicated antibodies: Keratin 10 (K10) (green), Ly6G (red). Scale bars, 100 μm. Data are representative of three independent experiments.

**Figure S11. Percentages and numbers of pDCs as well as MHC-II expression in B cells and cDCs in *Aire***^–/–^ **thymus.** (A) Percentages and numbers of thymic pDCs in *Aire*^+/+^ and *Aire*^–/–^ mice (*n* = 4). Data are shown as the mean and SE. Unpaired student’s t test was performed (*p<0.05, **p<0.01). (B) MHC-II expression in thymic pDCs, cDCs, and B cells from *Aire*^+/+^ and *Aire*^–/–^ mice examined by flow cytometry. MHC-II-positive percentages are shown. Data are representative of three independent experiments.

**Figure S12. Schematic representation of summary of this study.**
